# IL-17–driven tumor cell–intrinsic inflammatory programming creates an immunotherapy-permissive microenvironment

**DOI:** 10.1186/s12943-026-02726-2

**Published:** 2026-06-30

**Authors:** Kosuke Murakami, Shiki Takamura, Chiho Miyagawa, Shiro Takamatsu, Yoko Kashima, Koji Nagaoka, Yukari Kobayashi, Yoshiyuki Hakata, Shigeki Kato, Sachiyo Tsuji-Kawahara, Ding Nan, Ronald Chandler, Satoru Takahashi, Masaaki Miyazawa, Kazuhiro Kakimi, Noriomi Matsumura

**Affiliations:** 1 Department of Obstetrics and Gynecology, Kindai University Faculty of Medicine, 1-14-1, Miharadai, Minami-Ku, Sakai, Osaka 590-0197 Japan; 2 Laboratory for Immunological Memory, RIKEN Center for Integrative Medical Sciences, Yokohama, Japan; 3 Department of Immunology, Kindai University Faculty of Medicine, Sakai, Japan; 4 Department of Arts and Sciences, Kindai University Faculty of Medicine, Sakai, Japan; 5 Department of Obstetrics, Gynecology and Reproductive Biology, College of Human Medicine, Michigan State University, East Lansing, USA; 6 Department of Anatomy and Embryology and Laboratory Animal Resource Center in Transborder Medical Research Center, Institute of Medicine, University of Tsukuba, Tsukuba, Japan

**Keywords:** Clear cell carcinoma, CD4, IL-17, Immunotherapy, Ovarian cancer, RORC, NF-κB, Th17

## Abstract

**Background:**

While immune checkpoint inhibitors (ICIs) have failed to improve outcomes in unselected ovarian cancer populations, objective responses are observed in a minority of ovarian clear cell carcinoma (OCCC) cases, implying biological heterogeneity and a yet-undefined immunologically responsive subset within this histotype.

**Methods:**

We performed immunohistochemical profiling of tumor-infiltrating immune cells and analyzed transcriptomic data from human OCCC cohorts. Functional studies were conducted using an immunocompetent syngeneic OCCC mouse model to assess the effects of IL-17 on tumor cell inflammatory signaling, immune microenvironment remodeling, and responsiveness to immune checkpoint blockade, including single-cell RNA sequencing of tumor-infiltrating T cells.

**Results:**

OCCC exhibited an immune-sparse tumor microenvironment with relative enrichment of CD4⁺ T cells. *RORC* expression was elevated in OCCC but showed intertumoral heterogeneity. In the transcriptome data (*n* = 180), an *IL17A*^high^ subset (5%), enriched within the *RORC*^high^ fraction, exhibited a T cell–inflamed gene expression profile independent of microsatellite instability and tumor mutational burden, yet was not associated with survival. Mechanistically, IL-17 directly activated NF-κB–dependent inflammatory programs in OCCC tumor cells, inducing cytokines and chemokines involved in T-cell recruitment and activation. In the syngeneic model, IL-17 exposure increased intratumoral CD4⁺ and CD8⁺ T-cell infiltration and activation. Single-cell profiling further revealed expansion of Th17/Tfh-like CD4⁺ T cells and cytotoxic, non-terminally exhausted CD8⁺ T cells. Consistent with these changes, anti–PD-L1 therapy improved survival in Th17-biased partial chimera mice.

**Conclusions:**

IL-17–responsive, tumor cell–intrinsic inflammatory programming remodels the tumor immune microenvironment toward an immunotherapy-permissive state. These findings establish IL-17–responsive tumor cell inflammatory programming as a mechanistic axis shaping immune checkpoint sensitivity and provide a rationale for biomarker-guided immunotherapy strategies.

**Supplementary Information:**

The online version contains supplementary material available at 10.1186/s12943-026-02726-2.

## Background

Ovarian cancer remains the most lethal gynecologic malignancy worldwide [1]. Although ovarian clear cell carcinoma (OCCC) is relatively uncommon globally, it accounts for over 25% of ovarian cancer cases in Japan [1]. OCCC is characterized by intrinsic chemoresistance and is associated with poor clinical outcomes. Consequently, the development of effective therapeutic strategies for patients with recurrent or treatment-refractory OCCC remains a major unmet clinical need [2, 3].

Immune checkpoint inhibitors (ICIs) have markedly improved clinical outcomes for various solid tumors. Gene expression profiling and histopathological analyses have identified an immunoreactive subtype of high-grade serous ovarian carcinoma (HGSOC), the most common ovarian cancer subtype [4–6]. However, phase III clinical trials evaluating ICIs in ovarian cancer have thus far failed to demonstrate a clear clinical benefit [7–11]. In contrast, OCCC is generally considered an immune-cold tumor [12]. Nevertheless, the PEACOCC trial, a phase II study of previously treated advanced or recurrent gynecologic clear cell carcinoma, demonstrated objective clinical responses to immune checkpoint inhibition in a subset of patients [13]. Other evidence also suggests that OCCC, among ovarian cancer subtypes, may contain cases particularly amenable to immunotherapy [14, 15]. Together, these findings suggest that OCCC is not uniformly immunologically inert but rather comprises biologically heterogeneous tumors, including a distinct immunologically responsive subset.

OCCC has been reported to exhibit elevated expression of inflammatory cytokines, particularly interleukin-6 (IL-6), predominantly within the tumor compartment [16–18]. These findings suggest that OCCC may possess tumor cell–intrinsic inflammatory features distinct from other ovarian cancer histotypes. However, the relationship between such inflammatory tumor biology and the functional state of the tumor immune microenvironment (TIME)—specifically, under what conditions it renders an immune-sparse tumor permissive to checkpoint blockade—remains unclear. Addressing this gap is critical for defining biologically grounded biomarkers and rational immunotherapy strategies tailored to OCCC.

In this study, we integrated transcriptomic analyses of human OCCC with immunohistochemical profiling to define features of the TIME. We then used an immunocompetent syngeneic OCCC mouse model to functionally test how tumor cell–intrinsic inflammatory programming can remodel the TIME and shape responsiveness to immune checkpoint blockade.

## Methods

### Immunohistochemistry for human ovarian clear cell carcinoma samples

The KINDAI cohort comprised patients with OCCC who underwent primary surgical treatment at Kindai University Hospital between March 2019 and May 2020. All cases were diagnosed as OCCC by two board-certified gynecologic pathologists. Formalin-fixed, paraffin-embedded (FFPE) tumor specimens were sectioned at 3 μm and processed for immunohistochemical analysis. Single-color 3,3'-Diaminobenzidine (DAB) staining for CD68 (clone PG-M1, 1:100, #ab783, Abcam, Cambridge, UK) and CD163 (clone 10D6, 1:100, #NB110-59935, Novus Biologicals), as well as dual immunohistochemical staining for CD8 (clone 4B11, 1:250, #LCL-L-CD8-4B11, Leica Biosystems, Nussloch, Germany) and CD103 (clone EPR4166(2), 1:6000, #ab129202, Abcam), were performed manually according to a previously reported protocol [6]. Immunohistochemical staining for CD4 (clone SP35, prediluted, Roche, Basel, Switzerland), CD20 (clone L26, prediluted, Roche), CD138 (clone syndecan-1, 1:100, Dako), PD-L1 (clone 28–8, 1:100, Abcam), and IL-6 (clone 10C12, 1:50, Leica Biosystems) was performed using the BenchMark ULTRA System (Roche). For CD4 and CD20, antigen retrieval was conducted at pH 9.0 and 95 °C for 60 min, followed by incubation with the primary antibodies for 32 min and 16 min, respectively. CD138 staining was performed after antigen retrieval at pH 9.0 and 95 °C for 30 min, with a primary antibody incubation time of 32 min. For PD-L1 staining, antigen retrieval was performed at pH 9.0 and 95 °C for 60 min, followed by a 32-min incubation with the primary antibody. Detection was carried out using the OptiView DAB Universal Kit and OptiView Amplification Kit (Ventana Medical Systems, Oro Valley, AZ, USA). All immunohistochemically stained slides were digitized using the NanoZoomer Digital Pathology System (version 2.8.24; Hamamatsu Photonics K.K., Shizuoka, Japan). The number of positively stained cells was quantitatively evaluated at × 400 magnification using QuPath software (version 0.2.0). Heatmap generation and hierarchical clustering analyses were performed using R software (version 4.5.1).

### RNA sequencing and data analysis of human ovarian clear cell carcinoma

RNA sequencing data from the KYOTO cohort and the JGOG3025 cohort (JGOG3025-TR1 study, NCT03159572) were analyzed using raw gene expression data (FASTQ files). In the KYOTO cohort, the OCCC samples were derived predominantly from primary ovarian tumors (38/40), with the remaining 2 samples obtained from recurrent ovarian tumors. All 13 HGSOC samples were obtained from primary ovarian tumors, including 3 collected after neoadjuvant chemotherapy. In the JGOG3025 cohort, all analyzed tumors were primary ovarian tumors as specified in the study protocol. The JGOG3025-TR1 transcriptomic dataset was generated through our multicenter collaborative study and has been reported previously [19]. Adapter sequences and low-quality reads were removed from raw FASTQ files using Trim Galore (https://github.com/FelixKrueger/TrimGalore). The trimmed reads were aligned to the human reference genome (GRCh38) using STAR (version 2.6) (https://github.com/alexdobin/STAR), and gene-level read counts and expression levels were quantified using RSEM (version 1.3.1) (https://github.com/deweylab/RSEM) and expressed as transcripts per million (TPM). For downstream analyses, a value of 1 was added to each TPM value, followed by log2 transformation. Single-sample Gene Set Enrichment Analysis (ssGSEA) was performed using the ssGSEA module (version 10.0.1) implemented in the GenePattern open-source platform (https://cloud.genepattern.org/gp/pages/login.jsf) [20]. The OCCC signature was defined using the gene set previously reported as the *OCCC_Up* signature [16]. The T cell–inflamed GEP score was calculated according to previously published methods [21, 22]. Classification of MSI-high and TMB-high tumors was performed using the same criteria and analytical pipelines as described in prior reports [23].

### Collection and analysis of gene expression microarray data from human ovarian cancer

Publicly available gene expression microarray datasets were searched in the Gene Expression Omnibus (GEO) DataSets repository hosted by the National Library of Medicine (https://www.ncbi.nlm.nih.gov/). The search was conducted on August 1, 2022, using the search term “ovarian cancer,” with the following filters applied: organism “Homo sapiens,” entry type “Series,” and study type “Expression profiling by array.” This search yielded a total of 546 datasets. From these datasets, we manually selected those in which multiple ovarian cancer histologic subtypes, including OCCC and HGSOC, were analyzed within the same dataset. Only datasets containing at least four samples for each histologic subtype were included. Gene expression data from the selected datasets were downloaded from the GEO database and used for subsequent analyses. The GEO accession numbers and corresponding original publications for all publicly used datasets included in this analysis are provided in Supplementary Table 1. For data preprocessing, a value of 1 was added to each expression value followed by log2 transformation. The transformed data were then normalized within each dataset, and comparative analyses were performed across histologic subtypes.

### Establishment of an inbred ovarian clear cell carcinoma mouse model and cell lines

All mice were maintained under specific pathogen-free conditions at the Animal Facility of Kindai University. The OCCC mouse model, *Arid1a*^*fl/fl*^;(Gt)Rosa26Pik3ca^*H1047R^ on a CD-1 background, was generated as previously reported [24]. This mouse line was crossed onto a C57BL/6 background for eight generations using C57BL/6 wild-type mice (CLEA Japan, Inc., Meguro, Tokyo, Japan). Genomic DNA was extracted from tail biopsies, and genotyping was performed by PCR using primers previously reported [24] to confirm the genotype at each generation. As described in a previous report, ovarian clear cell carcinoma formation was induced by intra-ovarian bursal injection of adenovirus-Cre in mice aged 14–31 weeks [24]. Tumor tissues and ascites were dissociated, and tumor cells were isolated and cultured to establish three independent murine OCCC cell lines. These cells were maintained in Dulbecco’s Modified Eagle Medium (DMEM; high glucose, Thermo Fisher Scientific, Waltham, MA, USA) supplemented with 10% fetal bovine serum (FBS, CORNING, Corning, NY, USA) and 1% penicillin/streptomycin at 37 °C in a humidified incubator with 5% CO_2_. As a comparator, the ID8 cell line, which is widely used as a murine model of HGSOC, was purchased from Sigma-Aldrich (St. Louis, MO, USA). ID8 cells were cultured in RPMI-1640 medium (Thermo Fisher Scientific) supplemented with 10% FBS and 1% penicillin/streptomycin (Thermo Fisher Scientific) at 37 °C in a humidified incubator with 5% CO_2_. Cells were used for experiments after one or two passages.

### Mouse tumor inoculation models

The murine melanoma cell line B16 [25] was kindly provided by Dr. Hirotake Tsukamoto, and the murine lung carcinoma cell line LLC [26] by Dr. Kenji Chamoto, both from the Center for Cancer Immunotherapy and Immunobiology, Kyoto University. B16 cells (1 × 10^6^) or LLC cells (5 × 10^6^) were injected intradermally into C57BL/6 mice. Tumors were harvested 3 weeks after inoculation. Excised tumors were enzymatically digested with Collagenase D (1.25 mg/mL; Merck, Darmstadt, Germany) at 37 °C for 30 min. Mononuclear cells were then isolated using Percoll gradient centrifugation, stained with fluorochrome-conjugated antibodies, and subsequently analyzed by flow cytometry as described below.

### Culture of human ovarian clear cell carcinoma cell lines

The human ovarian cancer cell lines OVISE and RMG1, which have been reported to exhibit features characteristic of ovarian clear cell carcinoma (OCCC), were purchased from the Japanese Collection of Research Bioresources (JCRB) Cell Bank (Osaka, Japan). OVISE cells were cultured in RPMI-1640 medium (Thermo Fisher Scientific) supplemented with 10% FBS (CORNING) and 1% penicillin/streptomycin (Thermo Fisher Scientific), whereas RMG1 cells were maintained in Ham’s F-12 medium (Thermo Fisher Scientific) supplemented with 10% FBS (CORNING) and 1% penicillin/streptomycin (Thermo Fisher Scientific). All cells were cultured at 37 °C in a humidified incubator with 5% CO₂ and were used for experiments after one or two passages.

### RNA extraction and bulk RNA sequencing

Total RNA was extracted from three independent murine OCCC cell lines and the ID8 cell line using the RNeasy Mini Kit (Qiagen, Hilden, Germany) according to the manufacturer’s instructions. RNA quality was assessed, and only samples with an RNA integrity number (RIN) of 9 or higher were used for downstream analyses. RNA sequencing was performed by Novogene (Tokyo, Japan) or AZENTA (Burlington, MA, USA). Libraries were prepared using a non-stranded protocol with poly(A) selection, and sequencing was carried out on the NovaSeq platform (Illumina, San Diego, CA, USA).　Gene expression levels were quantified from the resulting FASTQ files using the Salmon pipeline [27] and expressed as TPM. For expression analyses, a value of 1 was added to each TPM value, followed by log2 transformation, and the transformed values were used for subsequent analyses. OCCC signature scores were calculated as described above. Hierarchical clustering analyses were performed using R software (version 4.5.1). Differentially expressed gene (DEG) analysis was conducted using the open-source web-based application iDEP (version 0.96; http://bioinformatics.sdstate.edu/idep96/) [28] in combination with R software (version 4.5.1). Gene Ontology enrichment analysis was performed using PANTHER (version 19.0).

### Multiplex cytokine assay

Three independent murine OCCC cell lines were seeded in 24-well plates at a density of 3 × 10^3^ cells per well. After 24 h of culture, recombinant mouse IL-17A protein, carrier-free (R&D Systems, Minneapolis, MN, USA; #421-ML-010/CF), was added to the culture medium at final concentrations of 1, 10, or 100 ng/mL. Cells were further incubated for 24 h, after which culture supernatants were collected. Quantification of murine cytokines was performed using the Bio-Plex Pro Mouse Cytokine GI 23-Plex Panel (Bio-Rad, Hercules, CA, USA; #M60009RDPD) according to the manufacturer’s instructions. For human cell line experiments, OVISE cells were seeded in 24-well plates at a density of 3 × 10^4^ cells per well. After 24 h of culture, recombinant human IL-17A protein, carrier-free (R&D Systems, Minneapolis, MN, USA; #317-ILB-050), was added to the culture medium at final concentrations of 1, 10, or 20 ng/mL. Culture supernatants were collected after 24 and 48 h of incubation. Human cytokine levels were quantified using the Bio-Plex Pro Human Cytokine GI 8-Plex Panel A (Bio-Rad; #M50000007A) according to the manufacturer’s protocol. All experiments were performed in triplicate.

### Reverse transcription-quantitative PCR

Complementary DNA (cDNA) was synthesized using the PrimeScript RT Reagent Kit (Perfect Real Time) (Takara Bio, Shiga, Japan) according to the manufacturer’s protocol. Reverse transcription was performed at 37 °C for 15 min, followed by enzyme inactivation at 85 °C for 5 s. Quantitative PCR reactions were prepared with a total volume of 20 μL per reaction, consisting of 10 μL of TB Green Premix Ex Taq II (2 ×) (Takara Bio), 0.4 μL of ROX Reference Dye (50 ×) (Takara Bio), 0.8 μL each of forward and reverse primers (10 μM), 6 μL of ultrapure water, and 2 μL of cDNA template. Reactions were dispensed into 96-well plates and analyzed using either the StepOnePlus Real-Time PCR System (Applied Biosystems, Waltham, MA, USA) or the Thermal Cycler Dice Real Time System (Takara Bio). *Tbp* was used as an internal control for murine cell lines, and *GAPDH* was used as an internal control for human cell lines. Each sample was analyzed in triplicate, and relative gene expression levels were calculated using the ΔΔCt method. Expression values for each sample were determined as the mean of ΔΔCt values obtained from triplicate measurements. The primer sequences used in this study are listed below.

- mouse *CXCL9*: forward, CCTAGTGATAAGGAATGCACGATG; reverse, CTAGGCAGGTTTGATCTCCGTTC.
- mouse *PDL1*: forward, TGCGGACTACAAGCGAATCACG; reverse, CTCAGCTTCTGGATAACCCTCG.
- mouse *TBP*: forward, CTACCGTGAATCTTGGCTGTAAAC; reverse, AATCAACGCAGTTGTCCGTGGC.
- human *IL17RA*: forward, CCATCAGCGAGCTAATGTCA; reverse, AATGGCGATGAGTGTGATGA.
- human *TNFA*: forward, CTCTTCTGCCTGCTGCACTTTG; reverse, ATGGGCTACAGGCTTGTCACTC.
- human *GAPDH*: forward, GTCTCCTCTGACTTCAACAGCG; reverse, ACCACCCTGTTGCTGTAGCCAA.

### siRNA-mediated NF-κB knockdown

Murine OCCC cells were seeded in 6-well plates at a density of 1 × 10^5^ cells per well and cultured for 24 h. After replacing the culture medium with Opti-MEM Reduced Serum Medium (Thermo Fisher Scientific; #31985062), siRNA transfection was performed using Lipofectamine 3000 Transfection Reagent (Thermo Fisher Scientific; #L3000001) according to the manufacturer's instructions. The siRNAs used included Silencer Select Negative Control siRNA (Thermo Fisher Scientific; #4390844) and Silencer Select siRNAs targeting NF-κB p65 (s72857 and s72858; #4390771, Thermo Fisher Scientific). Twenty-four hours after transfection, the medium was replaced with complete growth medium, and recombinant mouse IL-17A protein, carrier-free (R&D Systems, Minneapolis, MN, USA), was added at a final concentration of 10 ng/mL. After an additional 24 h of incubation, culture supernatants were collected, and total RNA was extracted using the RNeasy Mini Kit (Qiagen, Hilden, Germany) according to the manufacturer's protocol. Bulk RNA sequencing was performed as described above.

### Western blotting

Parental murine OCCC cell lines (without any transfection reagent or siRNA) were stimulated with recombinant mouse IL-17A (10 ng/mL) and harvested 5, 15, and 30 min after stimulation to capture phosphorylation events, with unstimulated cells processed in parallel as the baseline control; siRNA-transfected cells were prepared as described above. Protein lysates were prepared using Cell Lysis Buffer (Nacalai Tesque, Kyoto, Japan; #22352-04). Western blotting was performed using a wet transfer method. Protein samples were mixed with 4 × sample buffer containing 2-mercaptoethanol at a final ratio of 3:1 and denatured at 100 °C for 5 min. Denatured proteins were separated by SDS–PAGE using 5–20% gradient polyacrylamide gels (e-PAGEL, ATTO, Tokyo, Japan) and transferred to PVDF membranes using Towbin buffer at 90 V for 1 h. Membranes were washed with Tris-buffered saline containing Tween-20 (TBST) and blocked with 5% bovine serum albumin in TBST for 1 h at room temperature. Primary antibodies diluted in TBST were incubated with the membranes for 1 h at room temperature. The primary antibodies used were NF-κB p65 rabbit monoclonal antibody (clone D14E12, #8242, Cell Signaling Technology, Danvers, MA, USA), IκBα antibody (clone 44D4, #4812, Cell Signaling Technology), phospho-IκBα (Ser36) antibody (clone EPR6235(2), ab133462, Abcam, Cambridge, UK), and β-actin antibody (clone C4, #sc-47778, Santa Cruz Biotechnology, Dallas, TX, USA). After washing with TBST, membranes were incubated with the appropriate horseradish peroxidase–conjugated secondary antibodies (anti-rabbit IgG, HRP-linked antibody, #7074, Cell Signaling Technology; or rabbit anti-mouse IgG [H + L], HRP, #61–6520, Invitrogen, Waltham, MA, USA) for 1 h at room temperature. Immunoreactive bands were visualized using Immobilon Western Chemiluminescent HRP Substrate (Merck Millipore, Burlington, MA, USA) and imaged with the LAS-4010 imaging system (Cytiva, Marlborough, MA, USA). Band intensities were quantified using ImageJ software (National Institutes of Health, Bethesda, MD, USA).

### IL-17 treatment *in vivo*

Tumor establishment in mice was defined when any of the following criteria were met: (i) a body weight increase of ≥ 10% relative to baseline, (ii) a body weight gain of ≥ 2 g within one week, or (iii) the presence of obvious abdominal distension. Recombinant mouse IL-17A protein (carrier-free; R&D Systems, Minneapolis, MN, USA) was diluted in phosphate-buffered saline (PBS) to a final concentration of 1 µg per 100 µL. A total volume of 100 µL of the IL-17A solution was intravenously administered via the retro-orbital venous plexus under inhalation anesthesia. Control mice received 100 µL of PBS via the same route. Tumors were harvested 7 days after IL-17A administration and mechanically dissociated for subsequent analyses.

### Immunohistochemistry for mouse tumor samples

Ovarian tumor tissues were harvested from OCCC-bearing mice and processed into FFPE specimens. FFPE blocks were sectioned at a thickness of 4 µm, followed by antigen retrieval using Target Retrieval Solution (pH 9; DAKO) at 98 °C for 50 min. After blocking with Animal-Free Blocker (VECTOR Laboratories, Newark, CA, USA), tissue sections were incubated with an anti–NF-κB p65 antibody (clone D14E12, 1:100; Cell Signaling Technology, Danvers, MA, USA) for 30 min at room temperature. Sections were then incubated with Novolink Polymer (Leica Biosystems, Wetzlar, Germany) for 30 min at room temperature, and signal detection was performed using ImmPACT DAB (VECTOR Laboratories). Stained sections were digitized using the NanoZoomer Digital Pathology System (Hamamatsu Photonics, Shizuoka, Japan).

### Flow cytometry

Tumor tissues excised from mice were finely minced with scissors and enzymatically digested using Liberase TM (Sigma-Aldrich, St. Louis, MO, USA) at 37 °C for 30 min. Following enzymatic digestion, tissues were mechanically dissociated, and red blood cells were removed using RBC lysis buffer. After Fc receptor blocking, single-cell suspensions were stained with Zombie Aqua Fixable Viability Dye (BioLegend, San Diego, CA, USA) to exclude dead cells, followed by staining with fluorochrome-conjugated antibodies against the following surface markers: CD4 (BV421, 1:400), CD8a (BV785, 1:800), CD44 (APC, 1:800), CD69 (FITC, 1:100), TIGIT (PE, 1:200), PD-1 (PE/Cy7, 1:400), and CD45.1 (BUV737, 1:400) (all from BioLegend). For intracellular cytokine staining, tumor-derived cells were stimulated with phorbol 12-myristate 13-acetate (PMA) and ionomycin (both from Merck) for 5 h at 37 °C in the presence of Brefeldin A and monensin. Cells were then fixed and permeabilized using the Transcription Factor Staining Buffer Kit (TONBO Bioscience, San Diego, CA, USA), followed by intracellular staining with antibodies against IL-17A (clone TC11-18H10.1, 1:100; BioLegend) and IL-17F (clone 9D3.1C8, 1:100; BioLegend). Data acquisition was performed using a BD LSRFortessa flow cytometer (Becton, Dickinson and Company, Franklin Lakes, NJ, USA), and data were analyzed with FlowJo software version 10.8.1 (Becton, Dickinson and Company). To obtain absolute cell numbers, a defined weight of each tumor was enzymatically dissociated, and the entire resulting single-cell suspension was acquired in full on the flow cytometer without subsampling. The number of events recorded within each gate (live, Zombie Aqua–negative cells) was therefore taken directly as the absolute number of cells of that subset and was normalized to tumor weight (expressed as cells per gram of tumor tissue).

### Single-cell RNA sequencing

As described above, recombinant mouse IL-17A was administered to OCCC-bearing mice, and ovarian tumors were harvested 7 days after treatment. Due to the limited number of cells recoverable from individual tumors, tumor tissues from 4–5 mice per group were pooled for each single-cell RNA sequencing (scRNA-seq) sample. Tumor tissues were finely minced with scissors and enzymatically digested with Collagenase D (1.25 mg/mL; Merck) at 37 °C for 30 min. The resulting single-cell suspensions were stained with an anti-CD45 antibody (clone 30-F11, 1:800; BioLegend, San Diego, CA, USA) and 7-aminoactinomycin D (7-AAD, 1:200; BioLegend), and viable CD45⁺7-AAD⁻ cells were sorted using a BD FACSAria II cell sorter (BD Biosciences, San Jose, CA, USA). Sorted cells were loaded onto a BD Rhapsody Cartridge and processed using the BD Rhapsody Cartridge Reagent Kit (BD Biosciences) according to the manufacturer’s instructions. Single-cell cDNA synthesis was performed by Immunogeneteqs (Chiba, Japan) using the BD Rhapsody cDNA Kit (BD Biosciences). Whole Transcriptome Analysis (WTA) libraries were generated using the Immune Response Panel Mm and sequenced on a NovaSeq 6000 S4 flow cell (NovaSeq 6000 S4 Kit v1.5; Illumina, San Diego, CA, USA), with a sequencing depth of approximately 20,000 reads per cell. Preprocessing and analysis of single-cell RNA sequencing data were performed in accordance with previously published methods [29]. Briefly, raw reads were trimmed using Cutadapt v2.10 (10.14806/ej.17.1.200), annotated using BD Biosciences–provided Python scripts, and mapped to the reference transcriptome using Bowtie2 v2.4.2 [30]. Cell barcode information was added to BAM files, and read counts for each gene in each cell were calculated using mawk. Count data were integrated into a single-cell gene expression matrix and analyzed using Seurat v5 [31]. Cells expressing fewer than 200 genes or exhibiting a mitochondrial gene expression fraction greater than 5% were excluded. Data normalization and variance stabilization were performed using sctransform v2. Dimensionality reduction was conducted by principal component analysis (PCA), and Uniform Manifold Approximation and Projection (UMAP) was generated using the first 30 principal components. Clustering was performed with resolution parameters adjusted to achieve optimal separation of clusters. Putative doublets were identified and removed using DoubletFinder v2.0. The initial analysis was performed on all sorted viable CD45⁺ immune cells. After unsupervised clustering and cell-type annotation, cells annotated as T cells were extracted for subclustering and downstream analyses. Although clusters corresponding to B cells, macrophages, dendritic cells, and neutrophils were also identified in the initial dataset, myeloid, dendritic cell, and neutrophil populations were too small for robust statistical comparison between IL-17–treated and control groups, and B-cell populations showed no notable changes. Therefore, subsequent analyses were restricted to T-cell clusters, which are presented in this study.

### T cell transfer and anti-PD-L1 antibody treatment

A transgenic C57BL/6 mouse that constitutively expresses *RORγt* in T cells (*RORγt* Tg) was previously generated [32]. Splenocytes were isolated from *RORγt* Tg mice, and red blood cells were removed using RBC lysis buffer, followed by T-cell enrichment. *RORγt* Tg donor mice were CD45.1^+^, whereas recipient OCCC mice were CD45.2^+^, allowing discrimination between donor- and host-derived cells. For negative selection, splenocytes were incubated with biotin-conjugated antibodies against B220 (clone RA3-6B2), CD11b (clone M1/70), CD11c (clone N418), CD49d (clone DX5), and Ter119 (clone TER-119) (all from BioLegend, San Diego, CA, USA). Cells were subsequently labeled with Streptavidin Particles Plus-DM and T cells were enriched by collecting the negative fraction using the BD IMag Cell Separation System (BD Biosciences, San Jose, CA, USA). To generate partial T-cell chimeras, 2 × 10⁷ enriched T cells were adoptively transferred into recipient mice once weekly for four consecutive weeks. Ovarian tumor formation was induced in OCCC mice on a C57BL/6 background by intrabursal administration of adenovirus-Cre. For immune checkpoint blockade therapy, an anti–PD-L1 antibody (clone 10F.9G2; Bio X Cell, Lebanon, NH, USA) was administered intraperitoneally at a dose of 250 µg per injection, twice weekly for four weeks, starting seven weeks after adenovirus-Cre administration. Overall survival was evaluated using the onset of severe deterioration associated with ascites accumulation or a body weight increase of ≥ 20% from baseline as endpoint criteria.

### Statistical analysis

Statistical analyses were performed using GraphPad Prism version 8.2.0 (GraphPad Software, San Diego, CA, USA). Comparisons among three or more groups were conducted using ordinary one-way analysis of variance (ANOVA), whereas comparisons between two groups were performed using Student’s t-test or the Mann–Whitney U test as appropriate, based on the distribution of the data. Progression-free survival (PFS) and overall survival (OS) were analyzed using the Kaplan–Meier method, and differences between survival curves were assessed using the log-rank test. Correlations were evaluated using Pearson’s correlation coefficient or Spearman’s rank correlation coefficient, as appropriate and as indicated in the figure legends. These statistical tests were two-sided, and a *p* value < 0.05 was considered statistically significant. To account for multiple comparisons, *p* values were adjusted using the Benjamini–Hochberg false discovery rate correction or Bonferroni correction. Adjusted *p* values < 0.05 were considered statistically significant.

## Results

### OCCC is characterized by predominant infiltration of CD4⁺ T cells and M2-like macrophages

Among the 12 patients with OCCC in the KINDAI cohort, 11 cases (91.7%) were classified as International Federation of Gynecology and Obstetrics (FIGO) stage I, while the remaining case was stage II. To characterize the features of the TIME in OCCC, we first performed detailed immunohistochemical analyses using representative tumor sections.

**Fig. 1:**
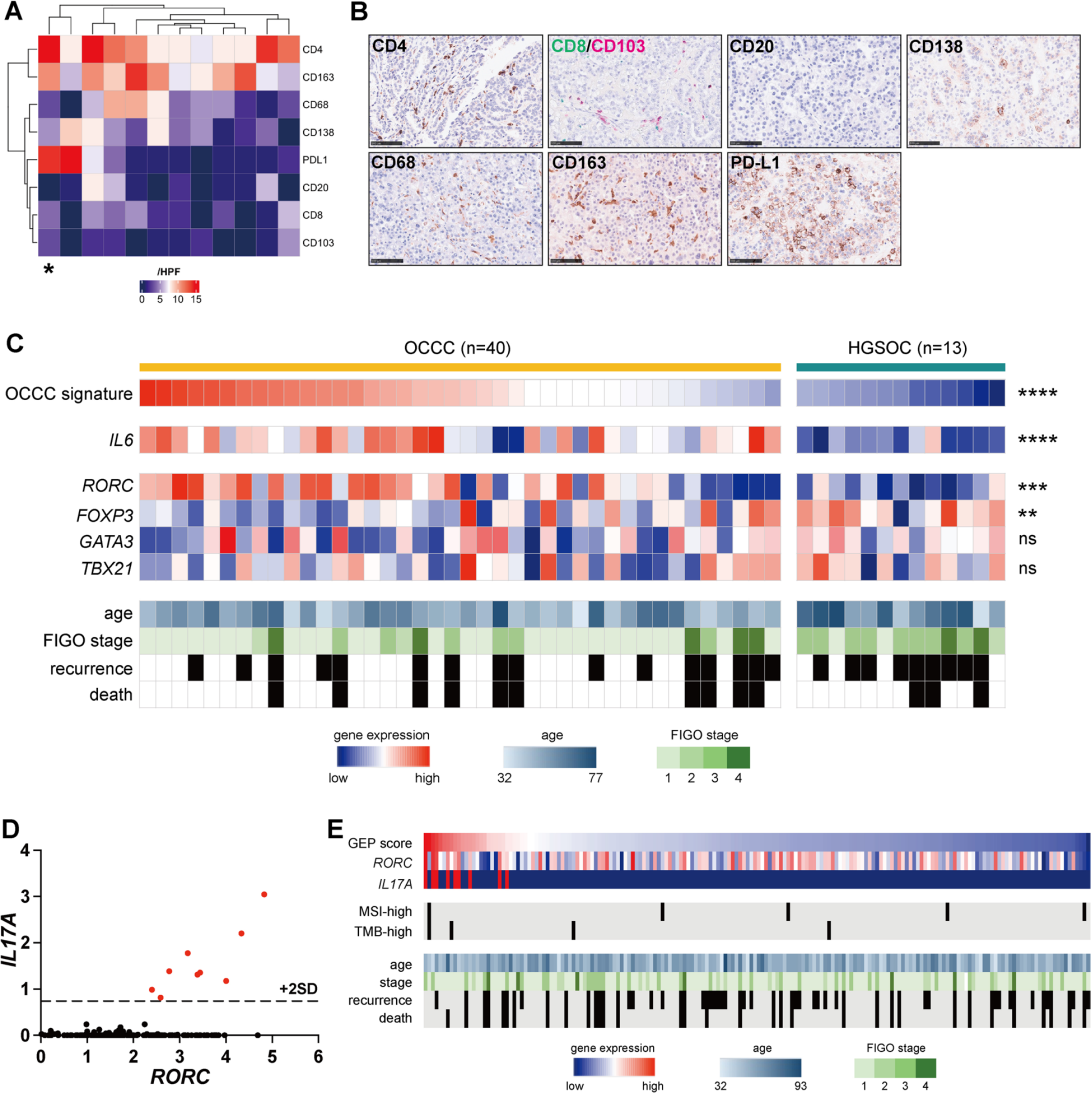
Ovarian clear cell carcinoma exhibits an immune-sparse but CD4⁺ T-cell–skewed microenvironment and contains an *IL17A*^high^ subset enriched within the *RORC*^high^ fraction with a T cell–inflamed transcriptional profile. **A** Immunohistochemical analysis of ovarian clear cell carcinoma (OCCC) tissues from 12 patients in the KINDAI cohort. CD8 and CD103 were evaluated by double staining, whereas CD4, CD20, CD68, CD138, CD163, and PD-L1 were assessed by single staining. The numbers of positive cells were quantified per high-power field (HPF, × 400) and visualized by heatmap clustering. An asterisk (*) indicates the representative case shown in (**B**). HPF, high-power field. **B** Representative immunohistochemical images of the case indicated by an asterisk in (**A**). Prominent infiltration of CD4⁺ T cells and CD163⁺ macrophages was observed, whereas CD8⁺ T-cell infiltration was scarce. Scale bar, 100 µm. **C** Bulk RNA sequencing analysis of tumor samples from 40 OCCC cases and 13 high-grade serous ovarian carcinoma (HGSOC) cases in the KYOTO cohort. Heatmap representation of the OCCC signature score calculated by single-sample gene set enrichment analysis (ssGSEA), gene expression levels of *IL6*, *RORC*, *FOXP3*, *GATA3*, and *TBX21*, and associated clinical information. Cases were first grouped by histologic subtype and then ordered within each subtype according to descending OCCC signature scores. Comparisons between OCCC and HGSOC were performed using the Mann–Whitney U test. OCCC, ovarian clear cell carcinoma; HGSOC, high-grade serous ovarian carcinoma; FIGO, International Federation of Gynecology and Obstetrics. **, *p* < 0.01; ***, *p* < 0.001; ****, *p* < 0.0001; ns, not significant. **D** Scatter plot illustrating the relationship between *IL17A* and *RORC* gene expression in 180 OCCC cases from the JGOG3025 cohort. Expression levels are shown as log_2_(Transcripts Per Million + 1). The horizontal dashed line indicates the threshold for *IL17A*^high^ status, defined as the cohort mean plus two standard deviations (+ 2SD). Red dots denote the nine tumors (5%) with markedly elevated *IL17A* expression, demonstrating their enrichment within the higher *RORC* expression spectrum. **E** Association of *IL17A* expression with the T cell–inflamed gene expression profile (GEP) score, immune checkpoint inhibitor (ICI)–related biomarkers (microsatellite instability status and tumor mutational burden), *RORC* expression, and clinical characteristics. *IL17A*^high^ cases exhibited significantly higher GEP scores compared with *IL17A*^low^ cases (*p* < 0.0001, Mann–Whitney U test). MSI, microsatellite instability; TMB, tumor mutational burden

Heatmap clustering based on quantitative immunohistochemical evaluation is shown in Fig. 1A, and representative immunohistochemical images are presented in Fig. 1B. Overall, intratumoral immune cell infiltration was sparse. However, where present, the infiltrate was characterized by a predominance of CD4⁺ T cells and CD163⁺ M2-like macrophages within the tumor stroma (Fig. 1A, B). The extent of CD4⁺ T-cell infiltration varied markedly among cases, ranging from pronounced stromal infiltration to minimal immune cell presence (Fig. 1A). In contrast, CD8⁺ T cells, including resident memory–like subsets with high antitumor activity (CD103⁺CD8⁺), CD20⁺ B cells, and CD138⁺ plasma cells were extremely rare (Fig. 1A, B). Consistent with this CD4-dominant immune infiltration pattern, most tumors lacked organized immune structures. Notably, a well-defined tertiary lymphoid structure was observed in 1 of 12 cases with predominant CD4⁺ T-cell infiltration. PD-L1–positive tumors were identified in only 2 of 12 cases (16.7%) (Fig. 1A), suggesting a tumor microenvironment with globally low IFNγ-dependent inflammatory activity.

OCCC is known to represent an immune-cold tumor characterized by limited intratumoral immune cell infiltration [12]. Our findings are consistent with previous reports.

### OCCC exhibits heterogeneous CD4⁺ T-cell–associated transcriptional programs, including a *RORC*^high^ subset

Gene expression profiles and associated clinical features derived from bulk RNA sequencing of tumor samples from the KYOTO cohort, comprising 40 OCCC and 13 HGSOC cases, are presented in Fig. 1C. The “OCCC signature,” defined as a gene expression profile characteristic of OCCC [16], was quantified in all 53 cases using single-sample Gene Set Enrichment Analysis (ssGSEA). OCCC tumors exhibited significantly higher OCCC signature scores than HGSOC tumors (*p* < 0.0001) (Fig. 1C). Consistent with previous reports [16, 33], *IL6* expression was markedly elevated in OCCC compared with HGSOC (*p* < 0.0001) (Fig. 1C).

Given the CD4⁺ T-cell–skewed immune features observed in OCCC, we next examined transcriptional programs associated with major CD4⁺ T-cell lineages. Evaluation of canonical lineage-defining transcription factors revealed that *RORC* was significantly upregulated in OCCC compared with HGSOC (*p* = 0.0005), whereas *FOXP3* was higher in HGSOC (*p* = 0.0099) (Fig. 1C). In contrast, expression levels of *TBX21* and *GATA3* did not differ significantly between OCCC and HGSOC (Fig. 1C). Notably, however, *RORC* expression displayed intertumoral heterogeneity within OCCC, suggesting that Th17-like programs characterize a distinct subset of tumors rather than the histotype ubiquitously. Correlation analysis across the four transcription factors further showed that *RORC* exhibited an inverse relationship with the other lineage-associated factors (Fig. 1C). Analyses of five publicly available ovarian cancer microarray datasets consistently demonstrated higher *RORC* expression in OCCC compared with other histologic subtypes (Supplementary Fig. 1).

Collectively, these findings suggest that the CD4^+^ T-cell–skewed TIME of OCCC is characterized by heterogeneous CD4^+^ T-cell–associated transcriptional programs, including a *RORC*^high^ subset that warrants further investigation in the context of the inflammatory immune microenvironment.

### A *RORC*^high^ subset of OCCC includes *IL17A*^high^ tumors with a T cell–inflamed transcriptional profile in a large clinical cohort

Building on the observation in the KYOTO cohort that OCCC included a *RORC*^high^ subset, we sought to determine whether tumors with higher *RORC* expression exhibit evidence of a Th17/IL-17–associated inflammatory immune contexture. To this end, we analyzed bulk RNA sequencing data from 180 OCCC cases in the JGOG3025 cohort—a large dataset of Japanese OCCC tumors established through our multicenter collaborative effort [19]. *IL17A* expression was markedly elevated in 9 cases (5%), exceeding the cohort mean by more than two standard deviations (Fig. 1D). Notably, *IL17A*^high^ cases were not uniformly distributed across the cohort; rather, they were enriched within the *RORC*^high^ subset, whereas the majority of tumors remained *RORC*^low^ (Fig. 1D), supporting heterogeneity in IL-17–associated immune contexture across OCCC.

In the KYOTO cohort, *IL17A* expression was near-background in most OCCC cases, precluding meaningful correlation analyses with *RORC* or *IL6* (Supplementary Fig. 2). In the JGOG3025 cohort, additional correlation analyses showed that *RORC* expression was modestly but significantly positively correlated with the OCCC signature score (*r* = 0.32, *p* < 0.0001), whereas no significant correlation was observed between *IL6* and *RORC* or between *IL6* and *IL17A* (Supplementary Fig. 3). Notably, *IL17A* expression showed a weak negative correlation with the OCCC signature score (*r* = − 0.24, *p* = 0.001), indicating that marked *IL17A* expression does not simply reflect the overall magnitude of the OCCC transcriptional program.

These *IL17A*^high^ tumors exhibited significantly higher T-cell–inflamed gene expression profile (GEP) scores—an indicator of intratumoral inflammation and T cell activation—compared with *IL17A*^low^ tumors (*p* < 0.0001; Fig. 1E) [34]. In contrast, *IL17A*^high^ status was independent of molecular features typically linked to immune-hot tumors and favorable ICI responsiveness, such as microsatellite instability–high (MSI-high) or high tumor mutational burden (TMB-high) (Fig. 1E). No characteristic gene mutations were identified in *IL17A*^high^ cases (Supplementary Fig. 4 A). In addition, there were no significant differences between *IL17A*^low^ and *IL17A*^high^ groups with respect to age and FIGO stage (Table 1). Furthermore, progression-free survival and overall survival did not differ significantly between the *IL17A*^low^ and *IL17A*^high^ groups (Supplementary Fig. 4B).

**Table 1:** Patient characteristics of JGOG3025 cohort

	number of samples	*IL17A* ^low^	*IL17A* ^high^	*P* value
Total	180			
Age (median 56 (32–93))				
> 56	87	83	4	0.81
≤ 56	93	88	5	
FIGO stage				
1	121	115	6	0.95
2–4	58	55	3	
MSI				
high	5	5	0	0.60
low	175	166	9	
TMB				
high	4	4	0	0.64
low	176	167	9	

FIGO stage was unavailable for one case

*MSI* Microsatellite instability, *TMB* Tumor mutational burden

Taken together, these findings indicate that an *IL17A*^high^ subset of OCCC—enriched within the *RORC*^high^ fraction—displays a T cell–inflamed transcriptional profile that is independent of MSI and TMB and is not prognostic in untreated disease, suggesting a potential link to responsiveness to immune checkpoint inhibition.

### A syngeneic OCCC mouse model recapitulates key tumor characteristics

**Fig. 2:**
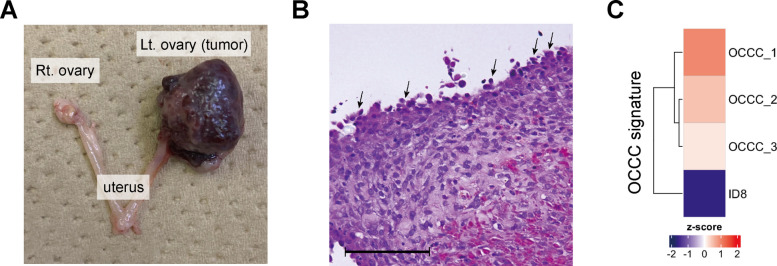
Establishment and characterization of an immunocompetent syngeneic OCCC mouse model. **A** Schematic overview of the generation of a syngeneic ovarian clear cell carcinoma (OCCC) mouse model. *Arid1a*^*fl/fl*^;(Gt)Rosa26Pik3ca^*H1047R^ mice were backcrossed onto a C57BL/6 background and ovarian tumorigenesis was induced by intrabursal administration of an adenovirus expressing Cre recombinase. Primary ovarian tumors accompanied by ascites formation and intraperitoneal dissemination were observed approximately 6–10 weeks after adenovirus-Cre injection. **B** Representative hematoxylin and eosin–stained sections of murine ovarian tumors. Tumors exhibited the characteristic hobnail cell morphology typically observed in human OCCC. Scale bar, 50 µm. **C** Comparison of OCCC signature scores between murine OCCC cell lines and the ID8 cell line. Based on bulk RNA sequencing data, single-sample gene set enrichment analysis was performed to calculate and normalize OCCC signature scores. Murine OCCC cell lines displayed gene expression profiles highly characteristic of OCCC compared with the ID8 high-grade serous ovarian carcinoma model

Next, to functionally interrogate the impact of the *IL17A*^high^ environment observed in human clinical specimens on antitumor immune responses, we established a syngeneic OCCC mouse model. In human OCCC, mutations in *ARID1A* and *PIK3CA* occur at high frequency [35]. The previous *Arid1a*^*fl/fl*^;(Gt)Rosa26Pik3ca^*H1047R^ OCCC mouse model was generated on a CD-1-background [24]. We backcrossed this model onto the C57BL/6 background for eight generations to establish a syngeneic strain amenable to genetic manipulation and adoptive immune cell transfer. Following administration of adenovirus-Cre into the ovarian bursa, ovarian tumors accompanied by peritoneal dissemination and ascites developed within approximately 6–10 weeks (Fig. 2A). Histopathological examination with hematoxylin and eosin staining revealed that the tumors displayed a characteristic hobnail pattern, a hallmark of human OCCC (Fig. 2B). Next, we analyzed immune cell infiltration in tumors and ascites. Consistent with observations in human OCCC, the overall number of infiltrating immune cells was low across all specimens, and Th17 cells were scarcely detected (Supplementary Fig. 5A-D). Collectively, these data demonstrate that this model recapitulates the fundamental features of immune-sparse OCCC under basal conditions.

We established three independent cell lines from cells recovered from murine OCCC tumors and ascites. Bulk RNA sequencing was performed on these cell lines, and OCCC signature scores were calculated using ssGSEA [16]. All murine OCCC cell lines exhibited higher OCCC signature scores than ID8 cells, a representative model of HGSOC, confirming their transcriptional similarity to OCCC (Fig. 2C).

### Tumor cell–intrinsic IL-17 signaling promotes an inflammatory cytokine milieu in OCCC

**Fig. 3:**
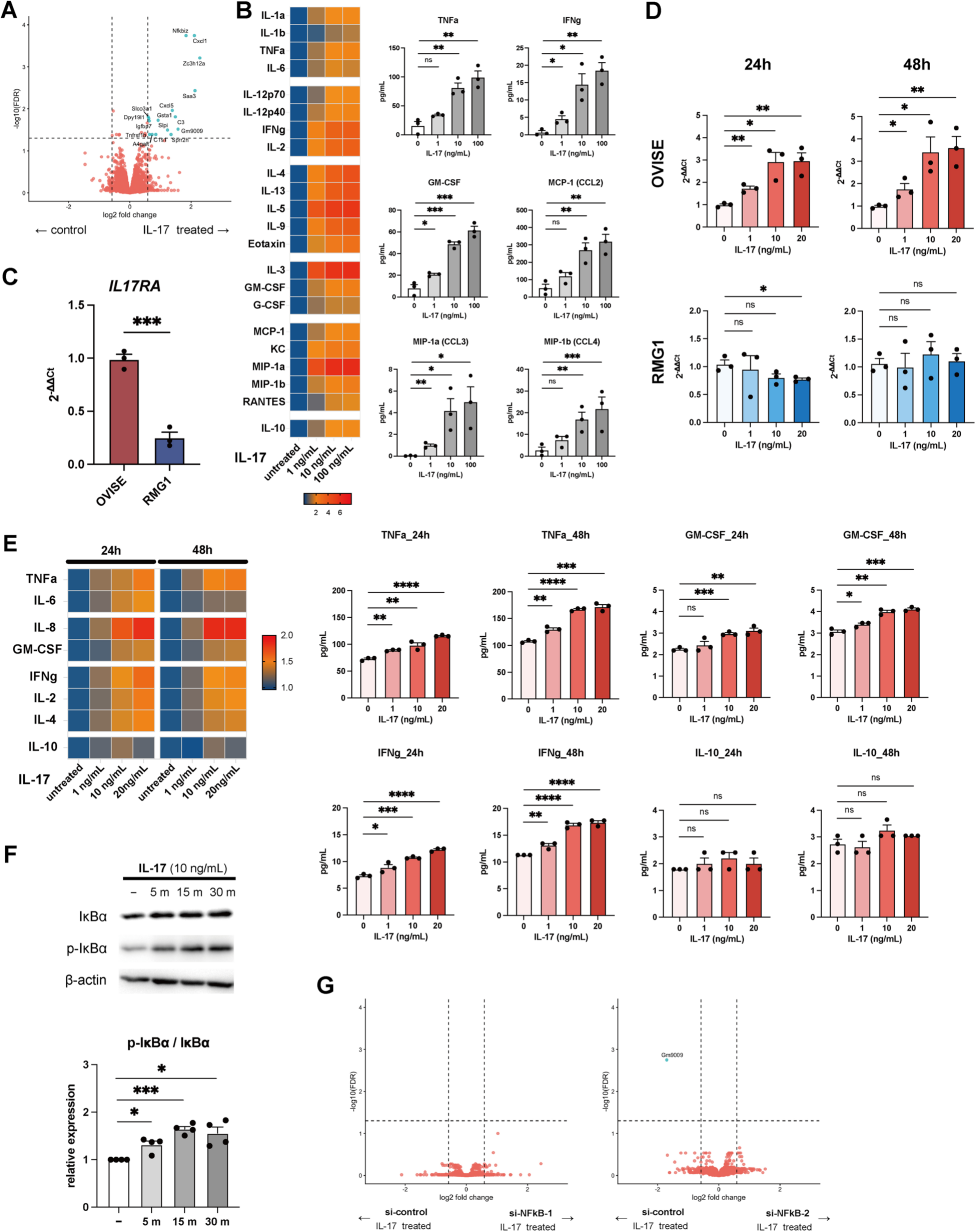
IL-17 directly acts on OCCC tumor cells to induce inflammatory cytokine production. **A** Differential gene expression analysis of murine OCCC cell lines following IL-17 stimulation. Genes significantly altered between control and IL-17–treated cells were defined by a false discovery rate (FDR) < 0.05 and |log₂ fold change|≥ 1.5. **B** Cytokine levels in culture supernatants of murine OCCC cell lines treated with IL-17. T cell–activating cytokines and T cell–recruiting chemokines, including CCL3 and CCL4, were upregulated in an IL-17 dose–dependent manner. The color bar indicates fold changes relative to untreated cells. *, *p* < 0.05; **, *p* < 0.01; ***, *p* < 0.001; ns, not significant; unpaired *t*-test. **C** *IL17RA* gene expression in human OCCC cell lines (OVISE and RMG1). ***, *p* < 0.001; unpaired *t*-test. **D** *TNFA* gene expression in human OCCC cell lines following IL-17 stimulation. In OVISE cells, *TNFA* expression increased in an IL-17 dose–dependent manner, whereas no such induction was observed in RMG1 cells. *, *p* < 0.05; **, *p* < 0.01; ns, not significant; unpaired *t*-test. **E** Multiplex cytokine profiling of OVISE cells treated with IL-17. Levels of T cell–activating cytokines, including TNFα, IFNγ, and GM-CSF, increased in a dose–dependent manner, whereas the immunosuppressive cytokine IL-10 remained unchanged. The color bar indicates fold changes relative to untreated cells. *, *p* < 0.05; **, *p* < 0.01; ***, *p* < 0.001; ****, *p* < 0.0001; ns, not significant; unpaired *t*-test. **F** NF-κB pathway activation in parental murine OCCC cells following IL-17 stimulation. Cells were left unstimulated (–) or harvested 5, 15, and 30 min after IL-17 (10 ng/mL) stimulation. (Upper) Representative Western blots of total IκBα, phospho-IκBα (Ser36), and β-actin. (Lower) Quantification of the phospho-IκBα/total IκBα ratio. Data are from four independent experiments (*n* = 4); error bars represent SEM. Each time point was compared with the unstimulated control by ratio paired *t*-test; *, *p* < 0.05; ***, *p* < 0.001. **G** Volcano plots showing differential gene expression in IL-17–stimulated OCCC cells transfected with control siRNA versus NF-κB–targeting siRNA. Two independent siRNAs (siNF-κB-1 and siNF-κB-2) were used to confirm reproducibility. Using significance thresholds of FDR < 0.05 and an absolute log₂ fold change ≥ 1.5, no biologically meaningful differentially expressed genes were identified

To investigate whether IL-17 directly acts on OCCC tumor cells and thereby influences the TIME, murine OCCC cell lines were treated with IL-17 and subjected to bulk RNA sequencing, which identified 16 genes that were significantly upregulated compared with control conditions (false discovery rate < 0.05 and |log₂ fold change|≥ 1.5) (Fig. 3A). Gene Ontology analysis of these genes revealed significant enrichment of pathways related to immune and inflammatory responses (Table 2).

**Table 2:** Gene ontology analysis

rank	adjusted *P* value	number of genes	GO Biological Process Pathways
1	4.13E-06	8	Response to bacterium
2	1.43E-05	5	Humoral immune response
3	1.43E-05	7	Immune effector process
4	1.43E-05	6	Regulation of immune effector process
5	3.04E-05	7	Inflammatory response
6	4.90E-05	4	Modulation of process of other organism
7	4.90E-05	7	Regulation of immune response
8	7.18E-05	8	Response to external biotic stimulus
9	7.18E-05	5	Adaptive immune response based on somatic recombination of immune receptors built from immunoglobulin superfamily domains
10	7.18E-05	8	Response to other organism
11	9.81E-05	6	Regulation of defense response
12	1.06E-04	8	Defense response
13	1.06E-04	8	Biological process involved in interspecies interaction between organisms
14	1.15E-04	5	Leukocyte mediated immunity
15	1.20E-04	8	Immune response

Corroborating these transcriptional alterations, analysis of conditioned media from murine OCCC cell lines treated with IL-17 revealed that IL-17 stimulation elicited a dose-dependent increase in the secretion of multiple cytokines. These included T cell–activating cytokines such as TNFα, IFNγ, and GM-CSF; the macrophage-recruiting chemokine MCP-1 (CCL2); and T cell–recruiting chemokines MIP-1α (CCL3) and MIP-1β (CCL4) (Fig. 3B).

To assess the conservation of this IL-17 responsiveness in human OCCC, we examined IL-17 responsiveness in human OCCC cell lines. Comparison of *IL17RA* expression between two representative human OCCC cell lines, OVISE and RMG1, revealed significantly higher *IL17RA* expression in OVISE cells (*p* = 0.0005) (Fig. 3C). Upon IL-17 stimulation, *TNFA* expression increased in a dose-dependent manner in *IL17RA*-high OVISE cells, whereas no clear induction was observed in RMG1 cells (Fig. 3D).

In human OCCC cells, we assessed a focused panel of T cell–relevant cytokines. In OVISE cells, IL-17 stimulation induced dose-dependent increases in the secretion of TNFα, GM-CSF, and IFNγ (Fig. 3E). In contrast, levels of the immunosuppressive cytokine IL-10 remained unchanged following IL-17 stimulation (Fig. 3E). Collectively, these data suggest that IL-17 acts directly on *IL17RA*-high OCCC tumor cells to foster an inflammatory cytokine milieu; in murine OCCC cells, this response also included T cell–recruiting chemokines.

Given that IL-17 is a known activator of NF-κB signaling [36–39], we next examined NF-κB pathway activation in response to IL-17 stimulation. In parental murine OCCC cells without transfection, IL-17 stimulation significantly increased the phospho-IκBα/total IκBα ratio (Fig. 3F), consistent with activation of canonical NF-κB signaling through IκBα phosphorylation. To determine whether NF-κB is functionally required for this response, we silenced NF-κB p65 using two independent siRNAs. Both siRNAs efficiently reduced NF-κB p65 levels (Supplementary Fig. 6) and attenuated IL-17–induced gene expression changes compared with control siRNA–transfected cells (Fig. 3G). These findings indicate that NF-κB plays a central role in the establishment of the tumor cell–intrinsic inflammatory transcriptional program induced by IL-17 stimulation.

### An IL-17^high^ tumor microenvironment enhances T cell infiltration and activation in OCCC

**Fig. 4:**
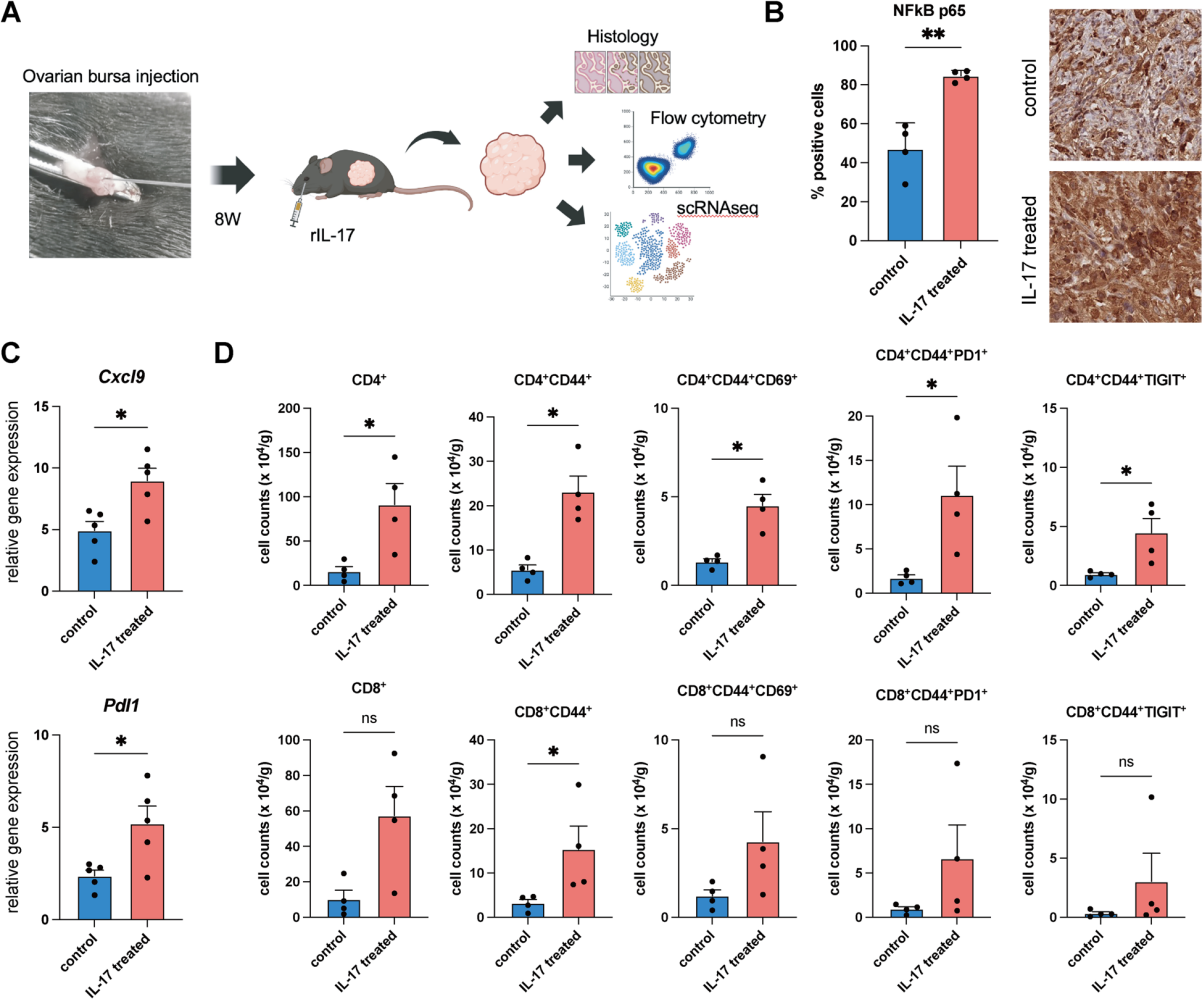
Systemic IL-17 administration induces an inflammatory shift in the tumor immune microenvironment and promotes T-cell infiltration and activation in vivo in OCCC. **A** Experimental scheme. Following induction of ovarian tumors by intrabursal administration of adenovirus-Cre in *Arid1a*^*fl/fl*^;(Gt)Rosa26Pik3ca^*H1047R^ mice, tumor-bearing mice were systemically treated with recombinant mouse IL-17A. Tumors were harvested one week later and subjected to immunohistochemistry (IHC), quantitative PCR (qPCR), flow cytometry, and single-cell RNA sequencing analyses. **B** Immunohistochemical analysis of NF-κB p65 expression in murine OCCC tumors. The proportion of NF-κB–positive cells was significantly higher in IL-17–treated tumors compared with control tumors. Error bars represent the standard error of the mean (SEM). **, *p* < 0.01, unpaired *t*-test. **C** Gene expression levels of *Cxcl9* and *Pdl1* in tumor tissues, assessed by qPCR. Relative expression levels were calculated using the ΔΔCt method and are presented as log₂ values normalized to housekeeping gene (*Tbp*) expression in normal mouse ovary. Error bars represent SEM. *, *p* < 0.05; unpaired *t*-test. **D** Flow cytometric analysis of tumor-infiltrating CD4⁺ and CD8⁺ T cells, their effector phenotype (CD44⁺), and activation marker expression (CD69, PD-1, and TIGIT). Absolute cell numbers were determined by acquiring the entire dissociated tumor suspension on the flow cytometer and were normalized to tumor weight. The y-axis indicates cell counts per gram of tumor tissue (× 10^4^/g). Error bars represent SEM. *, *p* < 0.05; ns, not significant; Mann–Whitney U test

To examine the effects of IL-17 on the TIME of OCCC *in vivo*, ovarian tumors were induced by intrabursal administration of adenovirus-Cre in *Arid1a*^*fl/fl*^;(Gt)Rosa26Pik3ca^*H1047R^ mice, and tumor development was confirmed based on macroscopic abdominal distension or body weight gain. Upon confirmation of established tumors, mice were intravenously injected with recombinant mouse IL-17A (Fig. 4A).

Analysis of tumors harvested one week after IL-17 treatment revealed that intratumoral expression of NF-κB p65 was significantly increased in IL-17–treated mice compared with control mice (*p* = 0.0015; Fig. 4B). Furthermore, analysis of immune-related gene expression revealed that *CXCL9*, a chemokine involved in T-cell recruitment, and *PDL1*, an IFNγ-inducible immune checkpoint molecule, were significantly upregulated in the IL-17–treated group (*p* = 0.021 and *p* = 0.013, respectively; Fig. 4C). Collectively, these data indicate that systemic IL-17 administration is associated with increased expression of chemokine and immune checkpoint markers within tumor tissues.

Next, tumor-infiltrating T cells were analyzed by flow cytometry. In the IL-17–treated group, the numbers of intratumoral CD44⁺ effector CD4⁺ and CD8⁺ T cells were significantly increased (Fig. 4D). Moreover, within the CD4⁺CD44⁺ T-cell compartment, the numbers of cells expressing activation markers indicative of antigen stimulation—including CD69, PD-1, and TIGIT—were all significantly increased (Fig. 4D). These findings imply that T cells recruited to the tumor undergo antigen-specific activation and expansion.

**Fig. 5:**
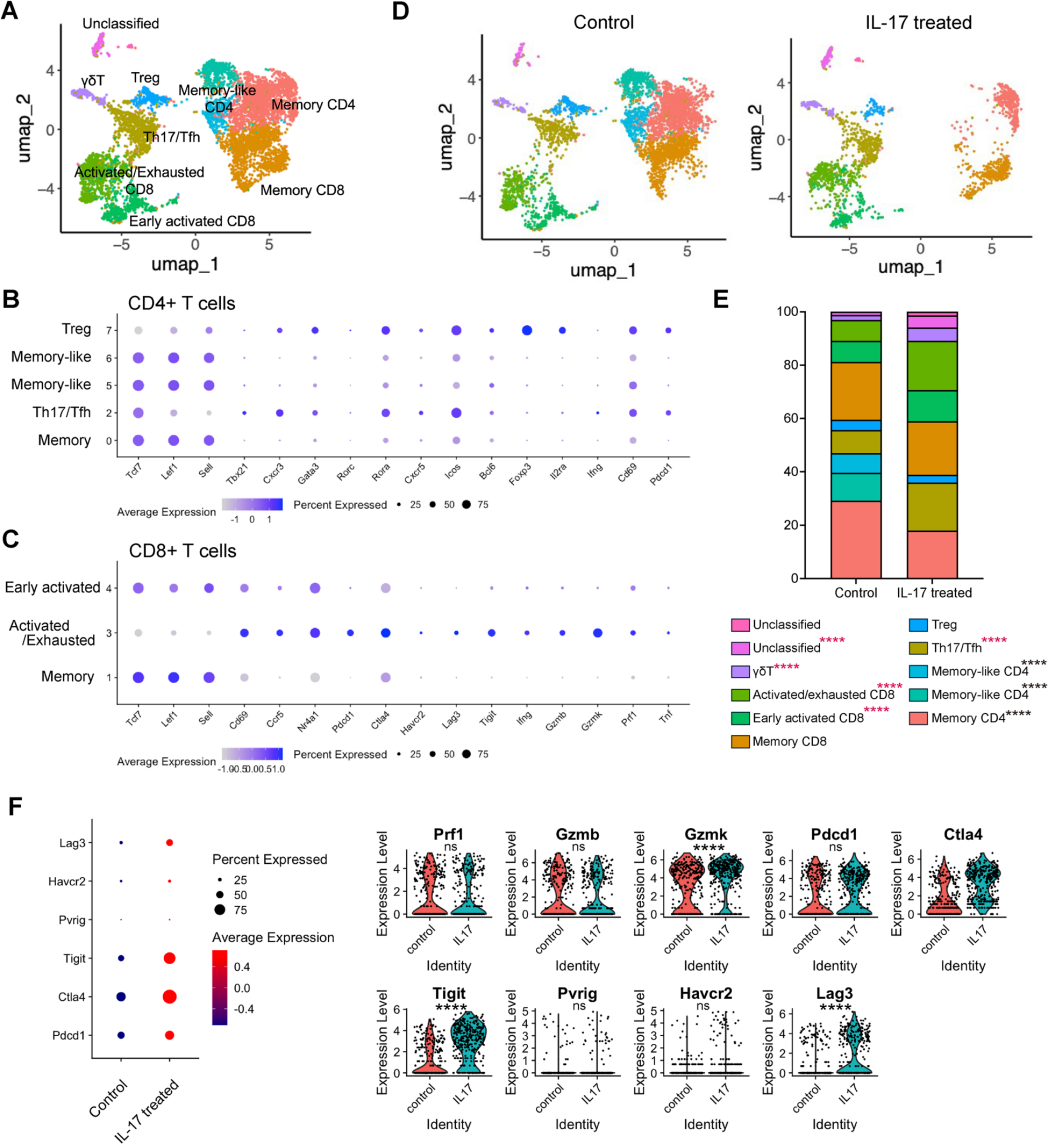
IL-17 administration reshapes the differentiation and functional states of tumor-infiltrating T cells. Tumor-infiltrating CD45⁺ cells used for single-cell RNA sequencing (scRNA-seq) were obtained by pooling tumors from 4–5 mice per group due to limited cell yield from individual tumors. Although the scRNA-seq dataset initially included all CD45⁺ immune cells, downstream analyses were restricted to T-cell populations because myeloid and B-cell populations were relatively scarce and did not permit robust statistical comparisons between groups. **A** Subclustering of tumor-infiltrating T cells. A total of 5,948 cells annotated as T cells were extracted and subjected to Uniform Manifold Approximation and Projection (UMAP) analysis, resulting in the identification of 11 distinct clusters. **B** Expression patterns of functionally relevant genes across CD4⁺ T-cell subsets. **C** Expression patterns of functionally relevant genes across CD8⁺ T-cell subsets. **D** Comparison of T-cell cluster composition between control and IL-17–treated tumors. **E** Quantitative analysis of the proportion of each T-cell cluster. The frequencies of Th17/Tfh CD4⁺ T cells and activated/exhausted CD8⁺ T cells were significantly increased in IL-17–treated tumors. ****, *p* < 0.0001; Fisher’s exact test with the Benjamini–Hochberg false discovery rate correction. Red asterisks indicate clusters increased in the IL-17–treated group, whereas black asterisks indicate clusters decreased in the IL-17–treated group. **F** Comparison of gene expression levels reflecting immune checkpoint molecule expression and cytotoxic activity in activated/exhausted CD8⁺ T cells between control and IL-17–treated groups. ****, adjusted *p* < 0.0001; ns, not significant; unpaired *t*-test with Bonferroni correction. Because the scRNA-seq samples were generated by pooling tumors from 4–5 mice per group, statistical comparisons in panels E and F reflect cell-level differences between pooled groups

To further characterize the functional properties of tumor-infiltrating immune cells induced by IL-17 treatment, we performed scRNA-seq on viable CD45⁺ immune cells isolated from tumor tissues. After clustering and cell-type annotation, T-cell populations were extracted for focused subclustering and downstream analyses. A total of 5,948 cells annotated as T cells were extracted and subjected to graph-based clustering, identifying 11 distinct clusters, including five CD4⁺ and three CD8⁺ T-cell clusters. The clusters were visualized using Uniform Manifold Approximation and Projection (UMAP) (Supplementary Fig. 7A–C). Based on the expression of signature genes, CD4⁺ T cells were classified into three major subsets: memory-type cells (*Tcf7*, *Lef1*, *Sell*), Th17/Tfh cells (*Rorc*, *Rora*, *Cxcr5*, *Icos*), and regulatory T cells (*Foxp3*, *Il2ra*) (Fig. 5A–C). In contrast, CD8⁺ T cells were categorized into memory-type cells (*Tcf7*, *Lef1*, *Sell*), early activated cells (*Cd69*, *Ccr5*, *Nr4a1*), and activated/exhausted cells (*Pdcd1*, *Havcr2*, *Lag3*, *Tigit*) (Fig. 5A–C). Comparison of cluster composition between IL-17–treated and control tumors revealed a marked reduction in the memory CD4⁺ T-cell compartment in the IL-17–treated group, accompanied by a significant expansion of the Th17/Tfh CD4⁺ T-cell compartment (Fig. 5D, E). These findings indicate that CD4⁺ T cells recruited into the tumor following IL-17 treatment undergo robust activation and preferentially differentiate toward Th17 or Tfh lineages, rather than adopting a conventional Th1-type differentiation program. Because IL-17 is generally considered an effector cytokine rather than a primary instructive signal for Th17 differentiation, the expansion of Th17/Tfh-like CD4⁺ T cells may reflect secondary cues within the tumor milieu, potentially including cytokine programs characteristic of OCCC. Consistent with this interpretation, the proportion of regulatory T cells did not increase following IL-17 treatment (Fig. 5D, E). In CD8⁺ T cells, IL-17 treatment resulted in a pronounced expansion of early activated and activated/exhausted clusters (Fig. 5D, E). Notably, activated/exhausted CD8⁺ T-cell clusters exhibited increased expression of immune checkpoint–related genes such as *Lag3* and *Tigit*, while maintaining expression of cytotoxic effector genes, including *Prf1*, *Gzmb*, and *Gzmk* (Fig. 5F). Thus, these profiles suggest that these cells do not exhibit features of terminal exhaustion, but rather retain substantial antitumor effector function.

Taken together, these findings demonstrate that IL-17 treatment remodels the TIME, establishing an environment enriched with activated and functionally competent CD4^+^ and CD8^+^ T cells. We postulated that such an immune contexture would confer heightened responsiveness to immune checkpoint inhibition.

### An IL-17^high^ tumor microenvironment enhances ICI responsiveness in OCCC

**Fig. 6:**
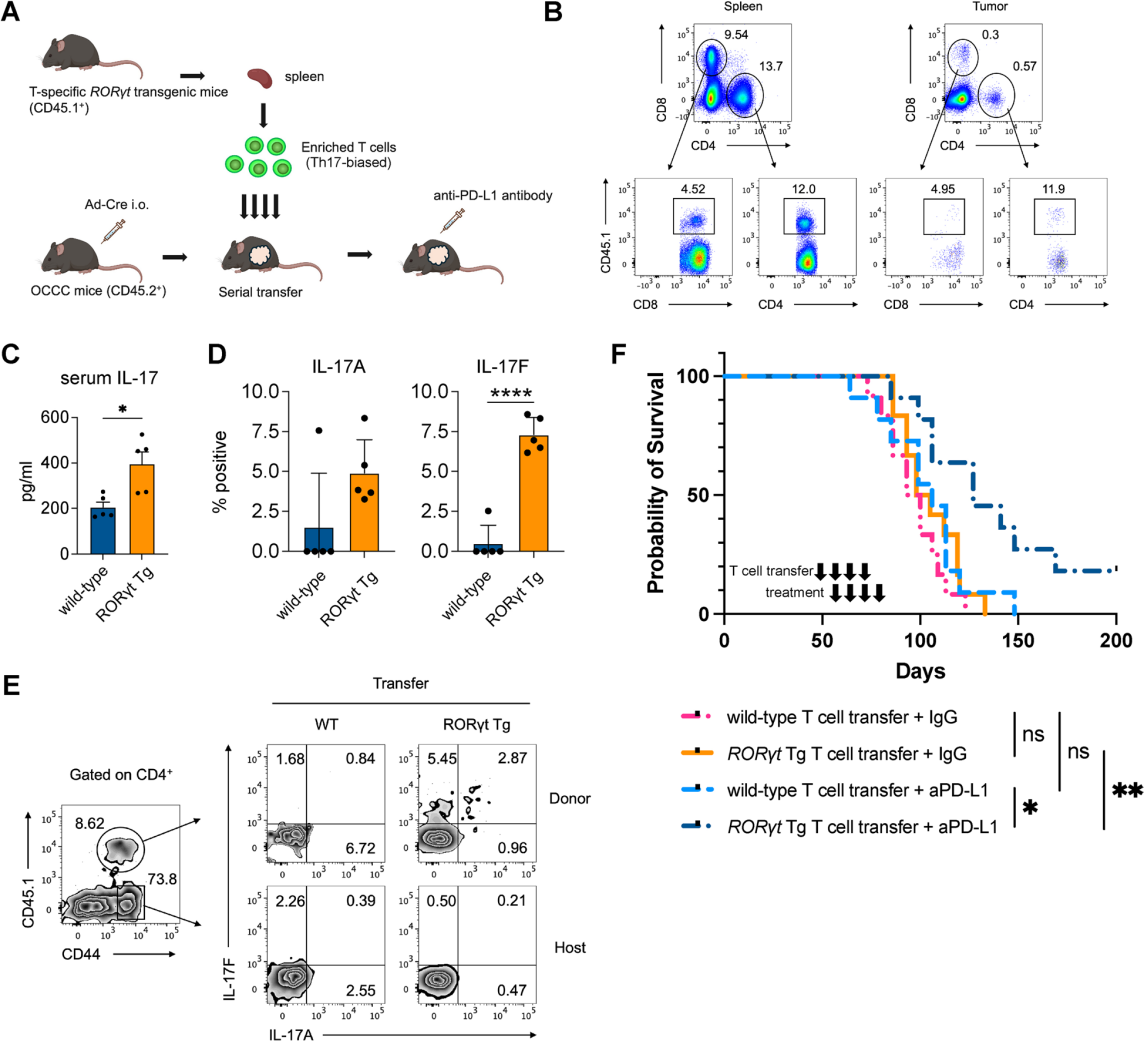
An IL-17^high^ tumor microenvironment enhances responsiveness to immune checkpoint inhibition in OCCC. **A** Schematic overview of the generation of partial chimeric mice to recapitulate a Th17-biased immune environment and the subsequent treatment strategy. CD45.1^+^ T cells derived from *RORγt* transgenic (*RORγt* Tg) or wild-type mice were repeatedly adoptively transferred into CD45.2^+^ tumor-bearing mice, followed by treatment with an anti–PD-L1 antibody. **B** Presence of *RORγt* Tg CD4⁺ T cells in *RORγt* Tg partial chimeric mice. Infiltration of *RORγt* Tg CD4⁺ T cells was detected in both the spleen and ovarian tumors. **C** Serum IL-17 levels in *RORγt* Tg partial chimeric mice and wild-type partial chimeric mice. **D** Proportion of intratumoral Th17 cells, defined as IL-17^high^ CD4⁺ T cells, within ovarian tumors. **E** IL-17 production by tumor-infiltrating CD4⁺ T cells following ex vivo stimulation. **F** Therapeutic efficacy of anti–PD-L1 antibody treatment in partial chimeric mice. Treatment with anti–PD-L1 antibody (clone 10F.9G2) administered intraperitoneally at 250 µg per injection, twice weekly for 4 weeks starting 7 weeks after adenovirus-Cre administration, significantly prolonged overall survival in mice receiving *RORγt* Tg T cells, whereas no survival benefit was observed in control conditions. Each group included 10 mice. Survival curves were generated using the Kaplan–Meier method and compared using the log-rank test. *, *p* < 0.05; **, *p* < 0.01; ns, not significant

We next sought to determine the impact of a Th17 signature on the therapeutic efficacy of immune checkpoint inhibition. To recapitulate a sustained Th17-biased immune environment in an OCCC mouse model, we generated bone marrow chimeric mice by transplanting bone marrow from *RORγt* transgenic (*RORγt* Tg) mice, in which T cells constitutively express *RORγt* and exhibit a Th17 bias [32], into *Arid1a*^*fl/fl*^;(Gt)Rosa26Pik3ca^*H1047R^ mice. However, intrabursal adenovirus-Cre administration in these chimeras resulted in premature mortality prior to tumor onset due to unknown causes. Consequently, we adopted an alternative strategy to generate "partial chimeric mice" by repeatedly adoptively transferring T cells isolated from the spleens of *RORγt* Tg or wild-type mice into the OCCC mouse model (Fig. 6A). In *RORγt* Tg partial chimeric mice, a defined proportion of *RORγt* Tg CD4⁺ T cells was detectable in the spleen (Fig. 6B). Serum IL-17 levels were significantly higher than those in partial chimeras generated by transfer of wild-type T cells (Fig. 6C). Crucially, these mice exhibited no overt signs of pathogenic autoimmunity. Moreover, infiltration of *RORγt* Tg CD4⁺ T cells into ovarian tumors was confirmed (Fig. 6B). When CD4⁺ T cells isolated from tumors were stimulated *ex vivo*, IL-17 production was not detected in host-derived or wild-type donor–derived T cells. In contrast, robust IL-17 production was observed in *RORγt* Tg donor–derived CD4⁺ T cells (Fig. 6D, E).

We leveraged these wild-type and *RORγt* Tg partial chimeric models to evaluate the therapeutic efficacy of anti–PD-L1 antibody treatment. Ovarian tumors were induced by intrabursal administration of adenovirus-Cre, and adoptive transfer of wild-type or *RORγt* Tg T cells was initiated. Beginning seven weeks after tumor induction, when tumor development was confirmed, mice were treated with anti–PD-L1 antibody twice weekly for four weeks (Fig. 6A). In wild-type partial chimeras, anti–PD-L1 treatment failed to confer a survival benefit compared with the control group (Fig. 6F). In *RORγt* Tg partial chimeric mice, which harbor tumors enriched with Th17 cells, overall survival was not prolonged in the untreated control group compared with wild-type partial chimeric mice (Fig. 6F). This finding indicates that, in the absence of immune checkpoint blockade, the Th17 signature does not influence survival outcomes, consistent with observations from the large human OCCC cohorts. However, anti–PD-L1 treatment of *RORγt* Tg partial chimeras resulted in a marked extension of overall survival compared with the corresponding control group (Fig. 6F).

Collectively, these data imply that while an IL-17–enriched tumor microenvironment is not prognostic during the natural course of disease, it constitutes a permissive immune state that sensitizes OCCC to immune checkpoint inhibition.

## Discussion

The secretion of IL-6 from tumor cells is one of the characteristics of OCCC [16–18, 33]. Given the established role of IL-6, together with additional cytokine cues, in promoting Th17 differentiation [40], it is plausible that tumor-intrinsic inflammatory programs characteristic of OCCC may contribute to the emergence of Th17-like transcriptional features in a subset of tumors. Notably, however, evidence of an *IL17A*^high^ state was observed in only a small fraction of OCCC tumors (~ 5%), and elevated *RORC* expression did not uniformly translate into high *IL17A* expression (Fig. 1). This suggests that a Th17-like transcriptional program does not necessarily equate to robust IL-17 production in all tumors. Additional cohort-level analyses further supported this distinction. In the larger JGOG3025 cohort, *RORC* expression was associated with the OCCC signature, whereas *IL17A* did not behave as a linear surrogate of either *IL6* or the broader OCCC transcriptional program. Together, these findings suggest that *RORC* captures a broader lineage-associated/inflammatory transcriptional state, whereas marked *IL17A* expression characterizes a more restricted subset within OCCC. The mechanisms underpinning this apparent dissociation between lineage-associated transcriptional features and cytokine output remain unclear and warrant further investigation. The systemic administration of IL-17 in our *in vivo* experiments was used as a controlled perturbation to isolate tumor-local inflammatory consequences of IL-17 signaling, rather than to recapitulate endogenous IL-17 dynamics in human OCCC. This reductionist approach enabled standardized evaluation of downstream TIME remodeling events and the functional impact of IL-17 on local tumor inflammatory responses.

IL-17 has been investigated as an inflammatory cytokine across a wide range of malignancies. In many cancer types, IL-17 has been implicated in tumor progression and angiogenesis, and is therefore often discussed as a negative prognostic factor [41, 42]. However, the biological role of IL-17 appears to be highly context dependent. In ovarian cancer, Kryczek et al. demonstrated that tumor-associated Th17 cells exhibit a polyfunctional effector phenotype, correlate positively with effector T-cell infiltration and negatively with regulatory T cells, and promote CXCL9 and CXCL10 production through cooperative signaling between IL-17 and IFNγ [43]. In that study, higher ascites IL-17 levels were associated with prolonged overall survival, supporting a protective role for Th17-associated immunity in ovarian cancer [43]. Our findings are consistent with this immunologic framework in that IL-17 exposure induced chemokine and cytokine programs in OCCC tumor cells and promoted T-cell infiltration and activation. At the same time, our data refine this concept in OCCC by showing that *IL17A*^high^ tumors constitute a small subset that is not prognostic in untreated disease, but instead appears to define an immune contexture permissive to immune checkpoint blockade.

Recent therapeutic studies further support this interpretation. Luo et al. reported that Th17-inducing dendritic cell vaccination remodeled the ovarian cancer tumor microenvironment and sensitized ID8 tumors to anti–PD-1 therapy, resulting in improved survival [44]. This strategy is now being clinically evaluated in combination with pembrolizumab in patients with advanced ovarian, fallopian tube, or primary peritoneal cancer [45]. Together, these observations suggest that IL-17/Th17-associated inflammation may represent an emerging immunologic axis capable of rendering selected ovarian tumor microenvironments more permissive to immune checkpoint blockade. Our study extends this concept to OCCC by identifying a tumor cell–intrinsic mechanism whereby IL-17 activates NF-κB-dependent inflammatory programming and thereby links Th17-associated immunity to immune checkpoint sensitivity.

Consistent with this model, our *in vitro* experiments demonstrate that IL-17 directly reprograms OCCC tumor cells in the absence of immune components, a process that likely cooperates with systemic immune effects observed *in vivo*. Based on these findings, we propose that the observed changes in tumor-infiltrating immune cells result from tumor cell–intrinsic responses to IL-17 via activation of the NF-κB pathway (Fig. 3). Consistently, flow cytometric analyses of the murine OCCC model revealed increased T cell infiltration under IL-17 treatment (Fig. 4), and subsequent scRNA-seq profiling demonstrated that these infiltrating T cells exhibited a mixed transcriptional state characterized by both activation markers and inhibitory molecules (Fig. 5). This immune phenotype is consistent with a tumor immune contexture poised for enhanced sensitivity to immune checkpoint blockade (Fig. 6). Collectively, these findings suggest that an IL-17/Th17-high TIME defines an immunologic state permissive to immune checkpoint inhibition and potentially exploitable across multiple solid tumors. Similar associations between IL-17/Th17-related programs and immune checkpoint responsiveness have also been reported in melanoma and renal cell carcinoma, suggesting that this biology may extend beyond ovarian cancer [46, 47].

We acknowledge several limitations in this study. First, the analyses of human OCCC specimens involved a relatively limited number of IL-17^high^ cases. Given the rarity of OCCC, prospective accrual of sufficiently large patient cohorts remains challenging. To mitigate this limitation, we strengthened the biological plausibility through functional validation. Nevertheless, prospective validation utilizing multicenter cohorts will be requisite to establish whether an IL-17^high^ tumor microenvironment can serve as a predictive biomarker for ICI responsiveness in OCCC. In addition, while we experimentally manipulated the IL-17 axis *in vivo*, the systemic IL-17A levels achieved in our models should be interpreted in context. Serum IL-17A levels were approximately 200 pg/mL in wild-type partial chimeras and 400 pg/mL in *RORγt* Tg partial chimeras. However, the intratumoral concentration and local bioavailability of IL-17 following systemic exposure were not directly quantified. Accordingly, the doses used for *in vivo* administration and *in vitro* stimulation were selected based on ranges commonly employed in prior studies, and the observed effects should be interpreted within this experimental (pharmacologic) context. How these circulating levels relate to intratumoral IL-17 activity and to clinically relevant exposures in patients remains to be clarified. Second, this study did not employ experimental systems that allow strict dissection of whether IL-17 acts directly on immune cells or indirectly through tumor cell–derived factors. By focusing on tumor cell–intrinsic responses to IL-17 stimulation and the subsequent remodeling of the TIME, we propose an “IL-17–tumor cell–immune microenvironment axis” as a mechanistic framework. Definitive causal inference, however, will necessitate future studies incorporating tumor cell–specific signaling manipulations and direct interrogation of IL-17 receptor expression and functional responses within immune cell populations.

In conclusion, our study identifies an IL-17–associated inflammatory state as a functional determinant of immune checkpoint sensitivity in OCCC, a clinically challenging and immunologically heterogeneous disease. We demonstrate that IL-17 directly reprograms tumor cells through NF-κB–dependent inflammatory signaling, reshaping the tumor microenvironment toward a T cell–inflamed, immunotherapy-permissive state independent of MSI [48] or TMB [49]. Rather than merely reflecting immune infiltration, IL-17 signaling defines a tumor-cell inflammatory state that actively shapes immune contexture. Beyond its clinical implications for OCCC, these findings support a broader principle whereby tumor cell–intrinsic inflammatory plasticity may contribute to immunotherapy responsiveness across tumor types.

## Supplementary Information

Supplementary Material 1: Supplementary Figure 1. *RORC* expression is elevated in OCCC compared with other ovarian cancer histologic subtypes. Comparison of *RORC* gene expression levels across ovarian cancer histologic subtypes using publicly available gene expression microarray datasets. Expression values were normalized within each dataset and visualized as dot plots. The GEO accession numbers and corresponding original publications for the datasets included in this analysis are provided in Supplementary Table 1. OCCC, ovarian clear cell carcinoma; HGSOC, high-grade serous ovarian carcinoma; OEC, ovarian endometrioid carcinoma; OMC, ovarian mucinous carcinoma; UD, undifferentiated carcinoma. *, *p* < 0.05; **, *p* < 0.01; ***, *p* < 0.001; ****, *p* < 0.0001; ns, not significant.

Supplementary Material 2: Supplementary Figure 2. Correlation analyses involving *IL17A* in the KYOTO OCCC cohort. (A) Correlation between *RORC* and *IL17A* expression in OCCC cases from the KYOTO cohort. (B) Correlation between *IL6* and *IL17A* expression in OCCC cases from the KYOTO cohort.*IL17A* expression was near-background in most cases, limiting meaningful correlation analysis in this cohort.

Supplementary Material 3: Supplementary Figure 3. Correlation analyses of *RORC*, *IL17A*, *IL6*, and the OCCC signature in the JGOG3025 cohort. (A) Correlation between the OCCC signature score and *RORC* expression. (B) Correlation between the OCCC signature score and *IL17A* expression. (C) Correlation between *IL6* and *RORC* expression. (D) Correlation between *IL6* and *IL17A* expression. Expression values are shown as log_2_(TPM + 1). Correlations were assessed using Spearman’s rank correlation coefficient.

Supplementary Material 4: Supplementary Figure 4. IL-17^high^ status is not associated with genomic alterations or survival outcomes in OCCC. (A) Somatic mutation profiles and IL17A expression levels in OCCC cases from the JGOG3025 cohort. For each case, mutated genes among the 50 genes included in the analysis are shown. Cases classified as *IL17A*^high^ are highlighted in red. (B) Comparison of progression-free survival (PFS) and overall survival (OS) between*IL17A*^high^ and *IL17A*^low^ groups in the JGOG3025 cohort. Survival analyses were performed using the Kaplan–Meier method, and differences between groups were assessed using the log-rank test. The x-axis represents time in days. PFS, progression-free survival; OS, overall survival; ns, not significant.

Supplementary Material 5: Supplementary Figure 5. The syngeneic OCCC mouse model does not exhibit a Th17 signature under baseline conditions. (A) Total number of infiltrating lymphocytes (cells passing the lymphocyte gate) in ovarian tumors, peritoneal dissemination lesions, and ascites in the OCCC mouse model under untreated conditions. (B) Comparison of total lymphocyte infiltration between the OCCC model and commonly used syngeneic tumor models (B16 melanoma and LLC lung carcinoma). Data are shown as total cell numbers per tumor (left) or normalized per tumor weight (right), as indicated. A similar trend was observed when CD4⁺ and CD8⁺ T-cell subsets were analyzed individually. (C) Representative gating strategy for flow cytometric analysis. Lymphocytes were first gated based on forward and side scatter properties, followed by singlet discrimination (FSC-A vs. FSC-H and SSC-A vs. SSC-H) and exclusion of dead cells. Subsequent analyses were performed on the resulting live, single-cell population. (D) Representative flow cytometry plots showing CD4⁺ and CD8⁺ T-cell distribution (upper panels) and intracellular IL-17A and IL-17F staining (lower panels) in spleen, ascites, ovarian tumors, and peritoneal dissemination lesions. Cells were stimulated ex vivo prior to intracellular cytokine staining. Data in panels A and B are from *n*= 4 mice per group; panels C and D show representative plots.

Supplementary Material 6: Supplementary Figure 6. siRNA-mediated knockdown of NF-κB p65 in murine OCCC cells. (A) Representative Western blots of murine OCCC cell lines transfected with control siRNA or two independent siRNAs targeting NF-κB p65 (si–NF-κB), with or without IL-17 stimulation. Both si–NF-κB constructs efficiently suppressed NF-κB p65 relative to control siRNA. (B) Quantification of NF-κB p65 band intensity normalized to β-actin, confirming efficient knockdown by both siRNAs. Error bars represent SEM. *, *p* < 0.05; **, *p* < 0.01; unpaired t-test.

Supplementary Material 7: Supplementary Figure 7. Subclustering of tumor-infiltrating T cells by single-cell RNA sequencing. (A) Uniform Manifold Approximation and Projection (UMAP) visualization of 5,948 cells annotated as T cells, extracted from single-cell RNA sequencing data. Cells were subdivided into 11 distinct clusters based on transcriptional profiles. (B) Feature plots showing the expression of *Cd4* and *Cd8a* across T-cell clusters. (C) Heatmap displaying the top five differentially expressed genes for each T-cell cluster.

Supplementary Material 8: Supplementary Table 1. Publicly available GEO datasets used for the cross-histotype RORC expression analysis.

## Data Availability

Bulk RNA sequencing data from the KYOTO cohort (GSE307291) and the JGOG3025 cohort (GSE306901) are available in the Gene Expression Omnibus (GEO) database. The murine bulk RNA sequencing data generated in this study have been deposited in GEO under accession number GSE319592. The single-cell RNA sequencing data generated in this study have been deposited in GEO under accession number GSE319959. All other data supporting the findings of this study are available from the corresponding author upon reasonable request. This study was also supported by the Nozawa Memorial Research Grant from the Japan Society of Gynecologic Oncology (to Kosuke Murakami).

## Abbreviations

DAB: 3,3'-Diaminobenzidine
DMEM: Dulbecco’s Modified Eagle Medium
FBS: Fetal bovine serum
FFPE: Formalin-fixed, paraffin-embedded
FIGO: International Federation of Gynecology and Obstetrics
GEP: Gene expression profile
GM-CSF: Granulocyte–macrophage colony-stimulating factor
HGSOC: High-grade serous ovarian carcinoma
ICIs: Immune checkpoint inhibitors
JCRB: Japanese Collection of Research Bioresources
MSI: Microsatellite instability
OCCC: Ovarian clear cell carcinoma
scRNA-seq: Single-cell RNA sequencing
ssGSEA: Single-sample Gene Set Enrichment Analysis
Tg: Transgenic
TIME: Tumor immune microenvironment
TMB: Tumor mutational burden
TPM: Transcripts per million
UMAP: Uniform Manifold Approximation and Projection
