# Supplementary figures and images for: IL-17–driven tumor cell–intrinsic inflammatory programming creates an immunotherapy-permissive microenvironment
Source: Mol Cancer. 2026 Jun 30;25:168. doi: 10.1186/s12943-026-02726-2 (PMC13348758; doi:10.1186/s12943-026-02726-2)

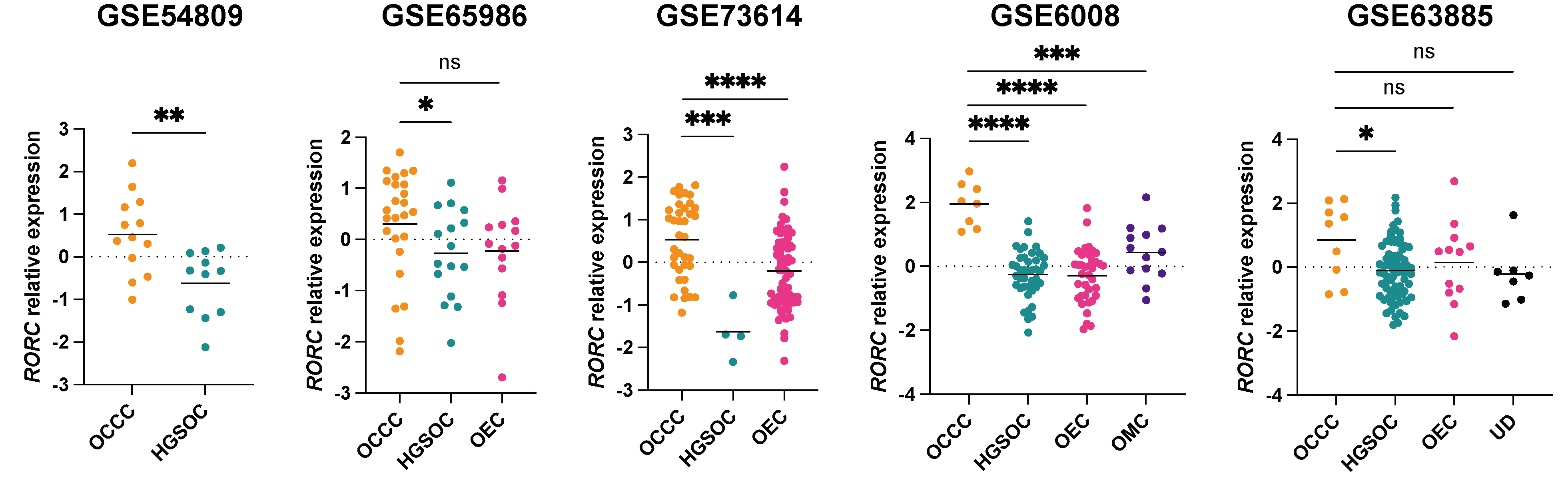

Supplement: Supplementary file 1 — Supplementary Material 1: Supplementary Figure 1. RORC expression is elevated in OCCC compared with other ovarian cancer histologic subtypes. Comparison of RORC gene expression levels across ovarian cancer histologic subtypes using publicly available gene expression microarray datasets. Expression values were normalized within each dataset and visualized as dot plots. The GEO accession numbers and corresponding original publications for the datasets included in this analysis are provided in Supplementary Table 1. OCCC, ovarian clear cell carcinoma; HGSOC, high-grade serous ovarian carcinoma; OEC, ovarian endometrioid carcinoma; OMC, ovarian mucinous carcinoma; UD, undifferentiated carcinoma. *, p < 0.05; **, p < 0.01; ***, p < 0.001; ****, p < 0.0001; ns, not significant. [file 12943_2026_2726_MOESM1_ESM.jpg]

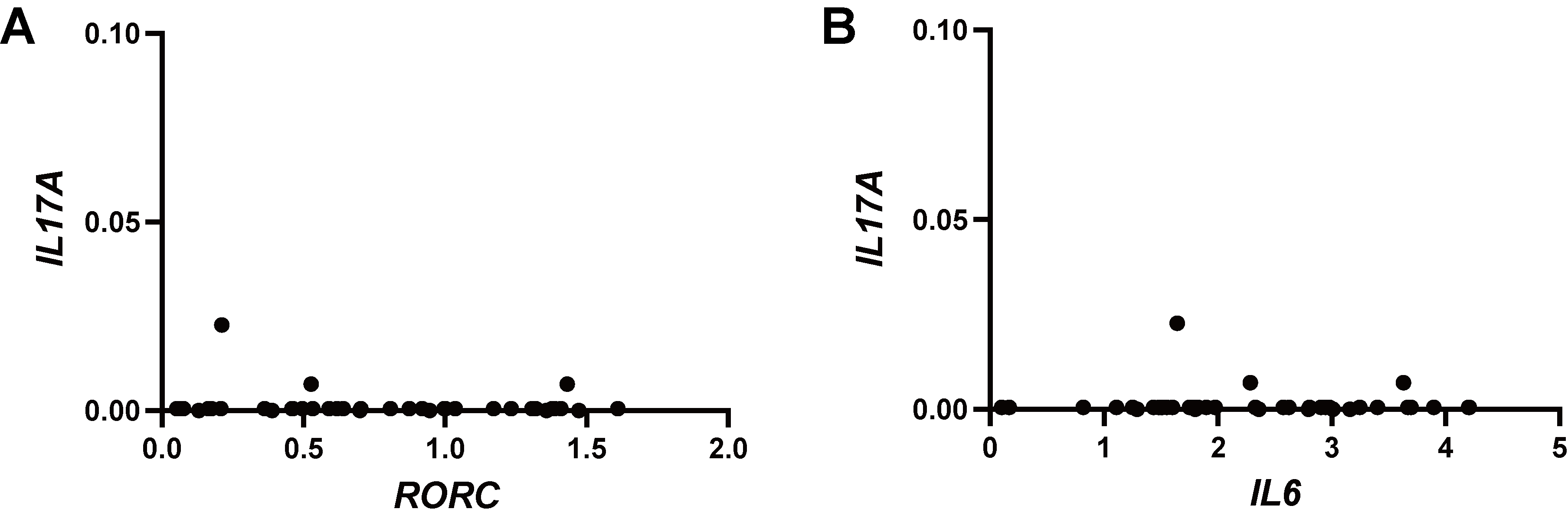

Supplement: Supplementary file 2 — Supplementary Material 2: Supplementary Figure 2. Correlation analyses involving IL17A in the KYOTO OCCC cohort. (A) Correlation between RORC and IL17A expression in OCCC cases from the KYOTO cohort. (B) Correlation between IL6 and IL17A expression in OCCC cases from the KYOTO cohort.IL17A expression was near-background in most cases, limiting meaningful correlation analysis in this cohort. [file 12943_2026_2726_MOESM2_ESM.jpg]

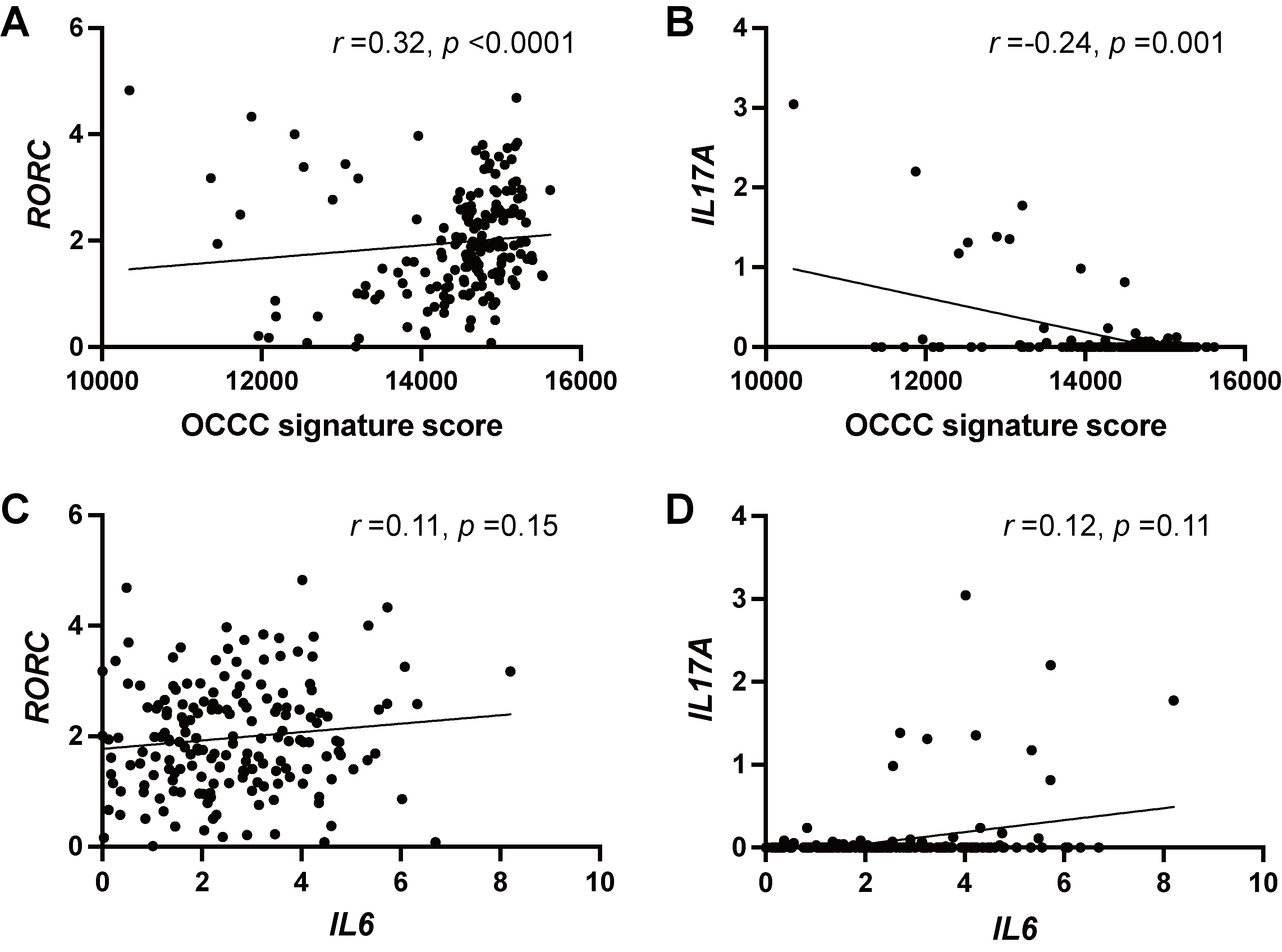

Supplement: Supplementary file 3 — Supplementary Material 3: Supplementary Figure 3. Correlation analyses of RORC, IL17A, IL6, and the OCCC signature in the JGOG3025 cohort. (A) Correlation between the OCCC signature score and RORC expression. (B) Correlation between the OCCC signature score and IL17A expression. (C) Correlation between IL6 and RORC expression. (D) Correlation between IL6 and IL17A expression. Expression values are shown as log2(TPM + 1). Correlations were assessed using Spearman’s rank correlation coefficient. [file 12943_2026_2726_MOESM3_ESM.jpg]

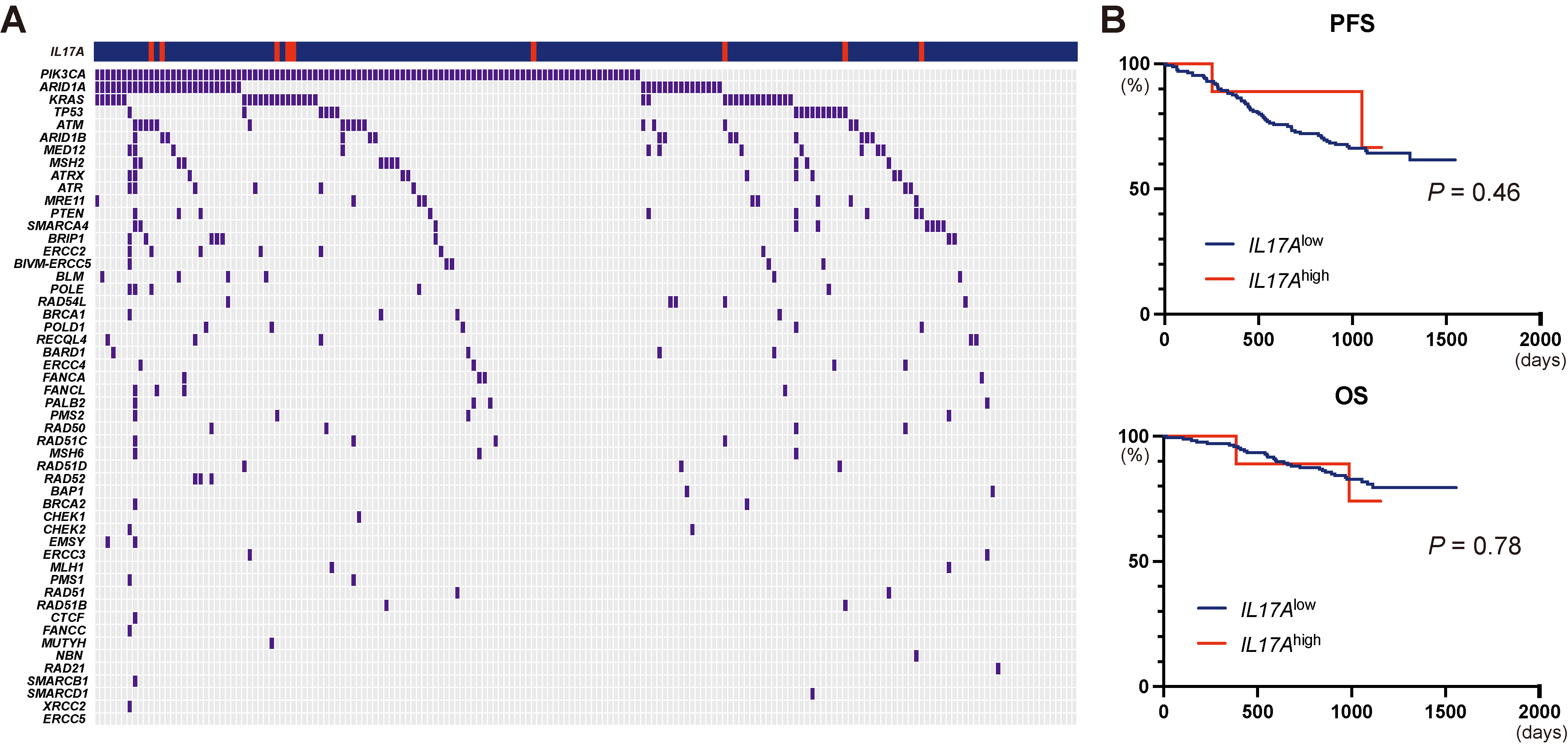

Supplement: Supplementary file 4 — Supplementary Material 4: Supplementary Figure 4. IL-17high status is not associated with genomic alterations or survival outcomes in OCCC. (A) Somatic mutation profiles and IL17A expression levels in OCCC cases from the JGOG3025 cohort. For each case, mutated genes among the 50 genes included in the analysis are shown. Cases classified as IL17Ahigh are highlighted in red. (B) Comparison of progression-free survival (PFS) and overall survival (OS) betweenIL17Ahigh and IL17Alow groups in the JGOG3025 cohort. Survival analyses were performed using the Kaplan–Meier method, and differences between groups were assessed using the log-rank test. The x-axis represents time in days. PFS, progression-free survival; OS, overall survival; ns, not significant. [file 12943_2026_2726_MOESM4_ESM.jpg]

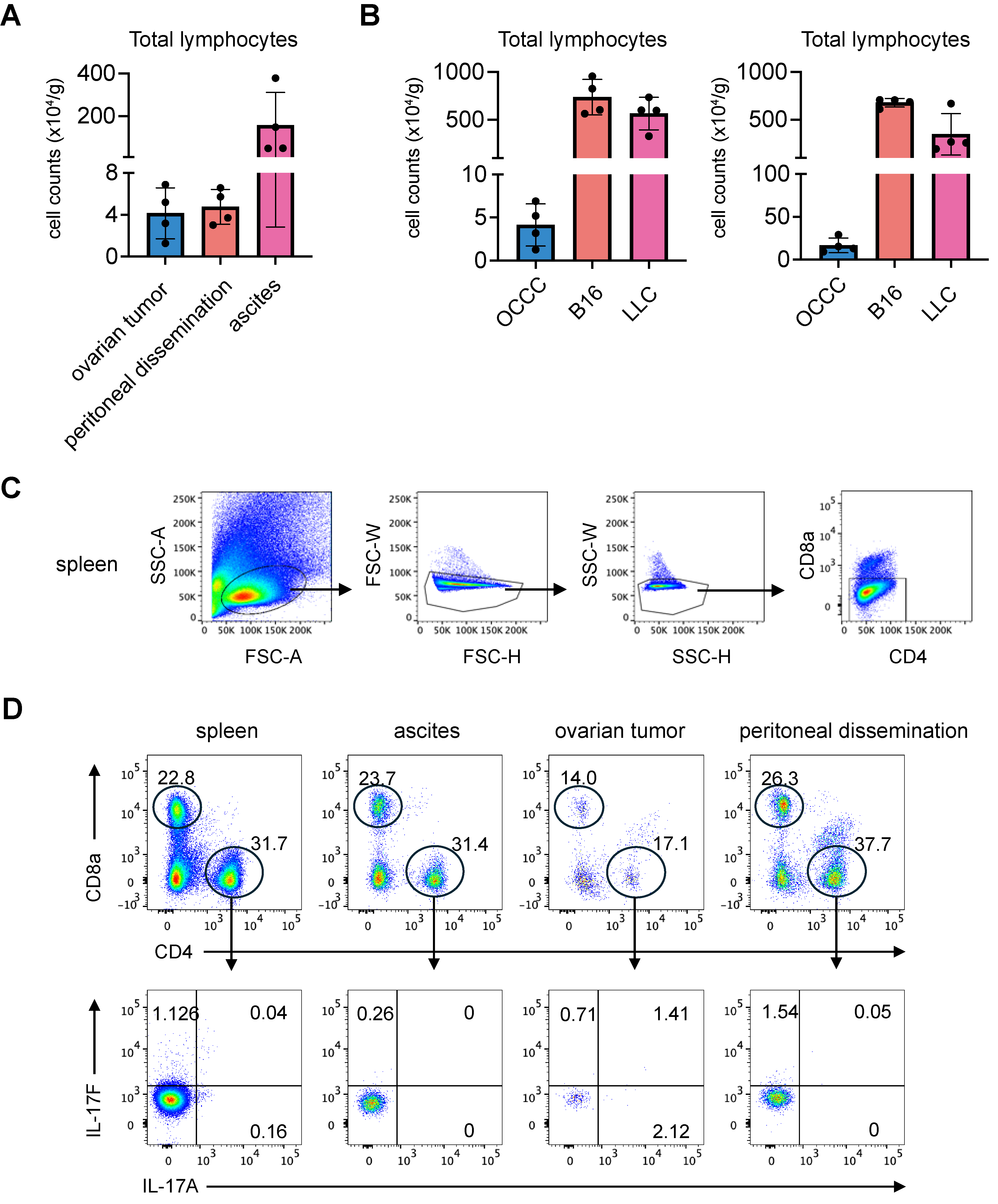

Supplement: Supplementary file 5 — Supplementary Material 5: Supplementary Figure 5. The syngeneic OCCC mouse model does not exhibit a Th17 signature under baseline conditions. (A) Total number of infiltrating lymphocytes (cells passing the lymphocyte gate) in ovarian tumors, peritoneal dissemination lesions, and ascites in the OCCC mouse model under untreated conditions. (B) Comparison of total lymphocyte infiltration between the OCCC model and commonly used syngeneic tumor models (B16 melanoma and LLC lung carcinoma). Data are shown as total cell numbers per tumor (left) or normalized per tumor weight (right), as indicated. A similar trend was observed when CD4⁺ and CD8⁺ T-cell subsets were analyzed individually. (C) Representative gating strategy for flow cytometric analysis. Lymphocytes were first gated based on forward and side scatter properties, followed by singlet discrimination (FSC-A vs. FSC-H and SSC-A vs. SSC-H) and exclusion of dead cells. Subsequent analyses were performed on the resulting live, single-cell population. (D) Representative flow cytometry plots showing CD4⁺ and CD8⁺ T-cell distribution (upper panels) and intracellular IL-17A and IL-17F staining (lower panels) in spleen, ascites, ovarian tumors, and peritoneal dissemination lesions. Cells were stimulated ex vivo prior to intracellular cytokine staining. Data in panels A and B are from n= 4 mice per group; panels C and D show representative plots. [file 12943_2026_2726_MOESM5_ESM.jpg]

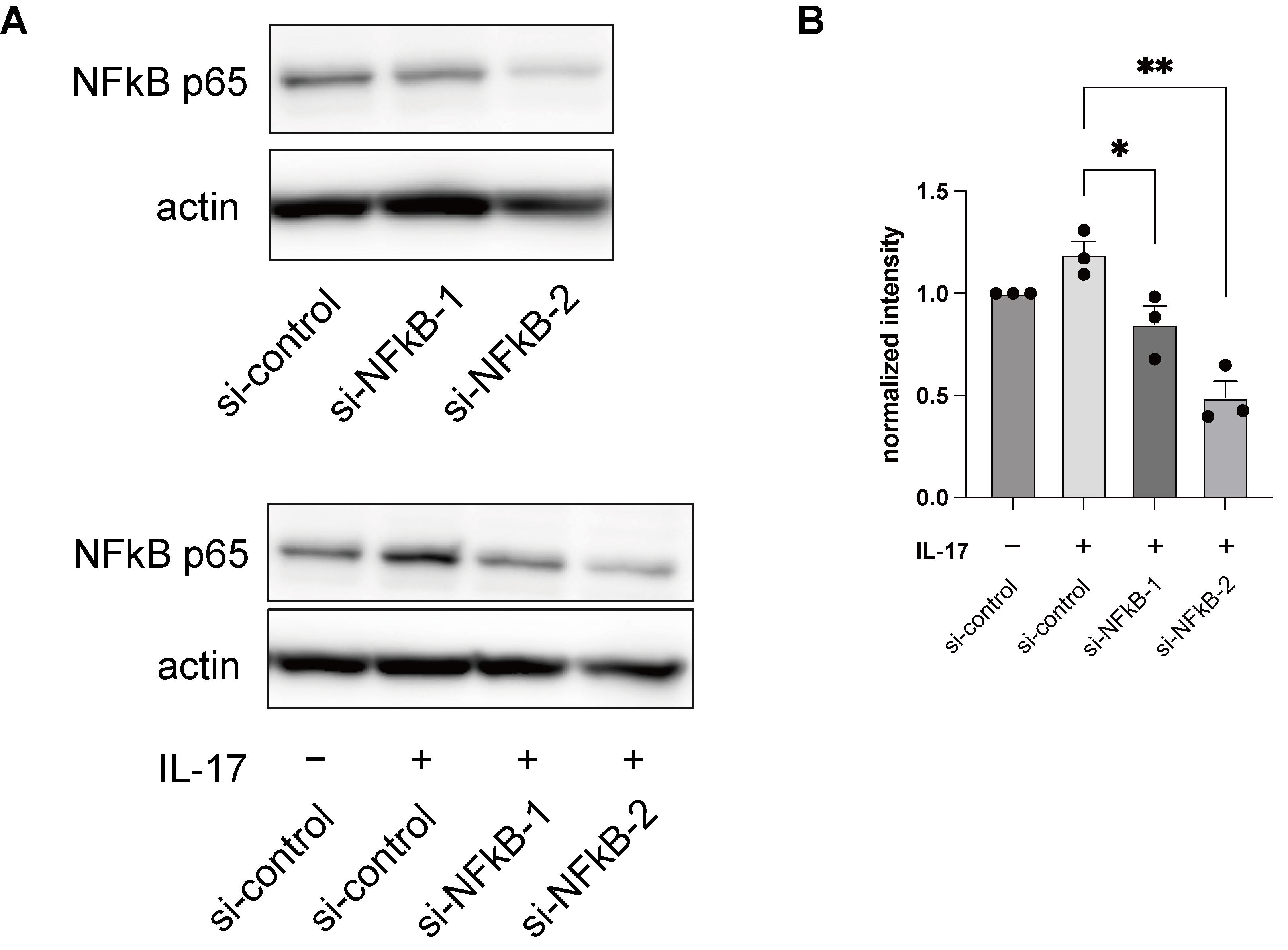

Supplement: Supplementary file 6 — Supplementary Material 6: Supplementary Figure 6. siRNA-mediated knockdown of NF-κB p65 in murine OCCC cells. (A) Representative Western blots of murine OCCC cell lines transfected with control siRNA or two independent siRNAs targeting NF-κB p65 (si–NF-κB), with or without IL-17 stimulation. Both si–NF-κB constructs efficiently suppressed NF-κB p65 relative to control siRNA. (B) Quantification of NF-κB p65 band intensity normalized to β-actin, confirming efficient knockdown by both siRNAs. Error bars represent SEM. *, p < 0.05; **, p < 0.01; unpaired t-test. [file 12943_2026_2726_MOESM6_ESM.jpg]

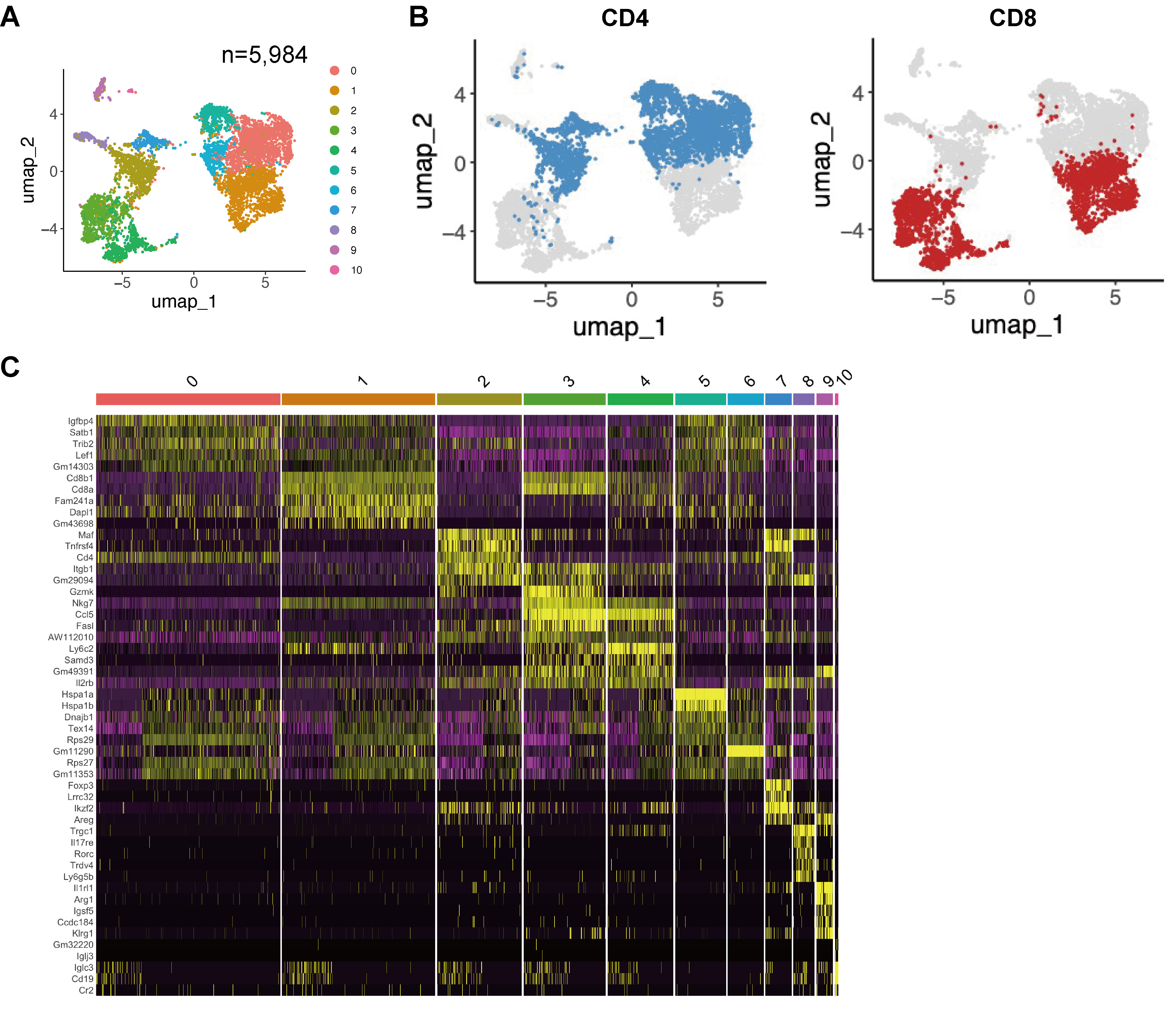

Supplement: Supplementary file 7 — Supplementary Material 7: Supplementary Figure 7. Subclustering of tumor-infiltrating T cells by single-cell RNA sequencing. (A) Uniform Manifold Approximation and Projection (UMAP) visualization of 5,948 cells annotated as T cells, extracted from single-cell RNA sequencing data. Cells were subdivided into 11 distinct clusters based on transcriptional profiles. (B) Feature plots showing the expression of Cd4 and Cd8a across T-cell clusters. (C) Heatmap displaying the top five differentially expressed genes for each T-cell cluster. [file 12943_2026_2726_MOESM7_ESM.jpg]
