# Supplementary material for: IL-17–driven tumor cell–intrinsic inflammatory programming creates an immunotherapy-permissive microenvironment
Source: Mol Cancer. 2026 Jun 30;25:168. doi: 10.1186/s12943-026-02726-2 (PMC13348758; doi:10.1186/s12943-026-02726-2)
Supplement: Supplementary file 8 — Supplementary Material 8: Supplementary Table 1. Publicly available GEO datasets used for the cross-histotype RORC expression analysis. [file 12943_2026_2726_MOESM8_ESM.pdf]

Supplementary Table 1. Publicly available GEO datasets used for the cross-histotype *RORC* expression analysis

GEO accession	First author	Journal	Year	Platform	Histology and Cases included in the present analysis	Notes
GSE54809	Tyekucheva S	J Mol Diagn	2015	Affymetrix Human Gene 1.0 ST Array	OCCC n = 13, HGSOC n = 11	Panel 1 of Supplementary Figure 1
GSE65986	Uehara Y	PLoS One	2015	Affymetrix Human Genome U133 Plus 2.0 Array	OCCC n = 25, HGSOC n = 16, OEC n = 14	Panel 2 of Supplementary Figure 1
GSE73614	Winterhoff B	Gynecol Oncol	2016	Agilent-014850 Whole Human Genome Microarray 4x44K G4112F	OCCC n = 37, HGSOC n = 4, OEC n = 66	Panel 3 of Supplementary Figure 1
GSE6008	Wu R	Cancer Cell	2007	Affymetrix Human Genome U133A Array	OCCC n = 8, HGSOC n = 41, OEC n = 37, OMC n = 13	Panel 4 of Supplementary Figure 1
GSE63885	Lisowska KM	Front Oncol	2014	Affymetrix Human Genome U133 Plus 2.0 Array	OCCC n = 9, HGSOC n = 73, OEC n = 12, UD n = 7	Panel 5 of Supplementary Figure 1

OCCC, ovarian clear cell carcinoma; HGSOC, high-grade serous ovarian carcinoma; OEC, ovarian endometrioid carcinoma; OMC, ovarian mucinous carcinoma; UD, undifferentiated carcinoma
